# Targeting USP22 reprograms the tumor microenvironment and sensitizes KRAS/p53-driven lung cancer to anti-PD-1 immunotherapy

**DOI:** 10.7150/thno.125898

**Published:** 2026-08-12

**Authors:** Keqiang Zhang, Ching Ouyang, Jonathan Castillo, Jinhui Wang, Walter Tsark, Yuanyuan Gao, Wendong Li, Minxiao Yang, Aimin Li, Britney Oeung, Colt Egelston, Jun Wu, Mahima Raul, Leonidas Arvanitis, Crystal Marconett, Dan J Raz

**Affiliations:** 1 Division of Thoracic Surgery, City of Hope National Medical Center, Duarte, California, USA.; 2 Integrative Genomics Core, City of Hope Comprehensive Cancer Center, Duarte, CA, USA.; 3 Department of Integrative Translational Sciences, City of Hope National Medical Center, Duarte, CA, USA.; 4 Transgenic/Knockout Program, City of Hope National Medical Center, Duarte, CA, USA.; 5 Pathology Core of Shared Resources, City of Hope National Medical Center, Duarte, CA, USA.; 6 Immuno-Oncology Facility, Department of Immune-Oncology, City of Hope National Medical Center, Duarte, CA, USA.; 7 Division of Comparative Medicine, City of Hope National Medical Center, Duarte, CA, USA.; 8 Department of Pathology, City of Hope National Medical Center, Duarte, CA, USA.

**Keywords:** Ubiquitin-specific peptidase (USP22), KRAS/p53-driven lung cancer, tumor microenvironment (TME), immune checkpoint inhibitors (ICI), anti-PD-1 therapy

## Abstract

**Rationale:**

Ubiquitin-specific peptidase 22 (USP22), a deubiquitinase and component of the “Death-from-Cancer” 11-gene signature, is overexpressed in multiple malignancies and linked to recurrence, therapy resistance, and poor prognosis. Its role in KRAS/p53-driven lung cancer and the response to immune checkpoint inhibitors (ICIs) remains poorly defined. Here, we investigated USP22 as a potential therapeutic target in KRAS/p53-driven lung cancer.

**Methods:**

A conditional Usp22 knockout (Usp22-KO) was generated in the KRAS^G12D^; p53^-/-^ (KP) mouse model. Cancer progression was monitored by micro-computed tomography (micro-CT). Multiplex immunofluorescence (mIF), RNA sequencing, and spatial transcriptomics profiled cancer and tumor microenvironment (TME) changes. Responses to anti-PD-1/PD-L1 therapies were compared between KP and Usp22-KO KP (KPU-) lung cancers.

**Results:**

USP22 was highly expressed in early-stage KRAS/p53-driven mouse lung cancers and strongly correlated with proliferation marker Ki67. Usp22 deletion suppressed cancer growth, prolonged survival, and promoted cancer differentiation. Spatial transcriptomics and mIF revealed reduced CD206+ M2 macrophages, myeloid-derived suppressor cells (MDSCs), TGF-β1, and angiogenesis, along with increased functional CD8+ T cells. Mechanistically, USP22 regulated gene expression and protein stability, reducing c-Myc, PD-L1, TGF-β1, and SPARC upon Usp22 loss. Compared with KP cancer, KPU- and SPARC-knockdown KP cancers showed reduced macrophage chemotaxis and impaired basal- and TGF-β1-induced M2 polarization of RAW264.7 cells, suggesting that TGF-β1 and SPARC downregulation partially contributes to decreased M2 macrophage infiltration in KPU- cancers. Notably, Usp22 loss enhanced the efficacy of anti-PD-L1 and anti-PD-1 therapies in orthotopic and subcutaneous KP lung cancer models, respectively. USP22 and SPARC expression were also strongly correlated in human lung cancers.

**Conclusions:**

USP22 promotes progression and immune evasion in KRAS/p53-driven lung cancer. Targeting USP22 reprograms the TME, suppresses oncogenic signaling, and sensitizes tumors to ICI, establishing USP22 as a promising therapeutic target.

## Introduction

Oncogenic KRAS mutations, found in about 25% of lung adenocarcinomas, are the most common drivers of non-small cell lung cancer (NSCLC). Targeting KRAS and its pathways has shown limited clinical success 1-3. KRAS-driven signaling pathways mainly promote carcinogenesis, while immune escape also plays a key role in disease progression 4. Currently, immune checkpoint inhibitors (ICIs) are central to systemic therapy with platinum-based chemotherapy as the current first-line treatment, but only about 20% of patients show modest, short-lived responses, largely due to the immunosuppressive tumor microenvironment (TME). Median overall survival for patients with advanced KRAS-mutant NSCLC remains low at 14 months 5, underscoring the urgent need for new therapies.

Ubiquitin-specific peptidase 22 (USP22), a member of the deubiquitinase (Dub) family, regulates key epigenetic modifications by catalyzing the removal of the mono-ubiquitin moiety from histone H2B (H2Bub) to control gene expression and cell-cycle progression 6. In cancer, USP22 was first identified as one of 11 “death-from-cancer signature” genes whose overexpression is strongly associated with disease recurrence, metastasis, and treatment failure in a wide range of cancer types 7. USP22 is frequently overexpressed in various cancers including lung, breast, and colon cancers and is associated with poor prognosis in cancer patients. Mechanistically, USP22 stabilizes multiple critical cancer drivers including c-Myc, cyclin D1 (CCND1), NAD-dependent protein deacetylase sirtuin-1 (SIRT1), and inhibits many p53 host-protective functions 8-10. Knockdown of USP22 in cancer cells often leads to cell-cycle arrest and decreased cancer growth and an enhanced response to chemoradiotherapy 10-12. In lung cancer, our studies show that knockdown of USP22 inhibits cancer stem cell (CSC) maintenance, angiogenesis, and xenograft tumor growth, while sensitizing cancer cells to cisplatin and radiation 13-15.

Of note, several studies further demonstrated that USP22 deubiquitinates and then stabilizes programmed death-ligand 1 (PD-L1), which mediates immune suppression of cancer cells 16, 17. Moreover, a recent study revealed that USP22 positively regulates Foxp3 activity in mouse regulatory T cells (Tregs) that suppress antitumor immune responses, and Treg-specific Usp22 knockout (Usp22-KO) suppressed the in vivo function of Tregs to improve antitumor immunity 18. Interestingly, the 1^st^ USP22 small-molecule inhibitor was shown to reduce Foxp3 in Tregs and suppress Tregs function to enhance host in vivo antitumor immunity 19. These studies demonstrate a potentially key role of USP22 in antitumor immunity and suggest that USP22 represents a promising target for immunotherapy to boost the antitumor immune response.

ICIs targeting programmed death-1 (PD-1) and its ligand PD-L1 have become the foundation of treatment for advanced KRAS-mutant lung cancer 12. Emerging evidence suggests that USP22 plays a critical role in modulating responses to these therapies. First, USP22 directly deubiquitinates and stabilizes PD-L1 protein on cancer cells, promoting immune evasion 16, 17. Second, USP22 enhances Foxp3 activity in Tregs, which suppress antitumor immunity; Treg-specific Usp22-KO impairs the in vivo function of Tregs 18. These mechanisms highlight USP22's dual role in supporting tumor immune evasion. In KRAS-mutant NSCLC, USP22 may play a particularly important role. Oncogenic KRAS fosters an immunosuppressive tumor microenvironment (TME) by stabilizing PD-L1 mRNA 20, driving inflammatory signaling and immune crosstalk that promote tumor growth, invasion, and progression 21. Elevated PD-L1 not only suppresses immune surveillance but also induces apoptosis of CD3+ T cells, contributing to therapy resistance 22, 23. Additionally, KRAS signaling facilitates the conversion of CD4+ T cells into Foxp3+ Tregs within the TME, which are required for lung tumorigenesis in KRAS-mutant mouse models 24. Consistently, a high density of Foxp3+ Tregs in human KRAS-mutant NSCLC correlates with increased recurrence and poor overall survival 25.

Depletion of USP22 has been shown to sensitize cancer cells to PD-L1-targeted immunotherapy, suggesting its inhibition could reverse TME-mediated immunosuppression 16, 17. The role of USP22 in immunosuppression, particularly in KRAS/p53-mutant lung cancer, remains unexplored in vivo. Clarifying its function in tumorigenesis and cancer-immune cell crosstalk is critical for developing USP22-targeted therapies for NSCLC. Given the importance of immune evasion in KRAS-driven lung cancer and USP22’s potential role in cancer progression and immune regulation, we hypothesized that targeting USP22 would suppress tumor growth and enhance response to ICI. Using a conditional USP22 knockout (Usp22-KO) in a KRAS^G12D^/p53^-/-^ (KP) lung cancer model, we observed reduced tumor burden, altered immune cell infiltration in the TME, and increased sensitivity to ICI. These findings highlight USP22 as a promising therapeutic target in KRAS-mutant NSCLC.

## Materials and Methods

### Usp22-KO in KP mice

Lox–Stop–Lox (LSL)* Usp22*-conditional knockout mice *(Usp22^Flox/Flox^*) were generated by *in vitro* fertilization with cryopreserved sperm (Usp22 Tm1a strain, MGI: 4364127, the Jackson laboratory) then crossed with (tm2(FLP*)Sor)/B6.129S4/J (Jax #012930) mice. *Usp22*-conditional knockout mice were further crossed with conditional *LSL-KRAS^G12D^-LSL-p53^Flox/Flox^* (KP, Jax #032435) mice to generate *KRAS*^G12D/+^-*p53^Flox/Flox^*-*USP22^Flox/Flox^* (KPU-) mice. The protocols for the care and treatment of mice were approved by the Institutional Animal Care and Use Committee of the City of Hope (IACUC-16005, -18138). Mice were housed at City of Hope Medical Center Animal Facility.

### Adenoviral Cre infection and pathological analysis of KP and KPU- lung cancer

A total of 36 KP and KPU*-* mice were inoculated intranasally with 1.0 × 10^7^ infectious particles in 40 μL of adenovirus expressing Cre recombinase (Ad-Cre, University of Iowa Viral vector core) with calcium phosphate coprecipitates to initiate lung cancer, as described previously 26, 27. The time course of cancer development, progression, and survival outcomes in KP and KPU- lung cancers were assessed using a combination of histological, molecular, and imaging analyses, including micro computed tomography (Micro-CT). For immune infiltration analysis, following Ad-Cre treatment, mice (6 - 8 weeks old; 6 males and 6 females per genotype) were euthanized at 10 - 12 weeks post-infection. The remaining mice were monitored for survival analysis.

### Immunohistochemistry and multiplex immunofluorescence staining

Mouse lungs were formalin-fixed, paraffin-embedded (FFPE), sectioned, and stained with hematoxylin and eosin (H&E). Immunohistochemistry (IHC) analysis of Ki67, CD31, cleaved caspase-3, and USP22 on mouse and human lung cancer tissues was performed, and USP22 IHC staining was scored as negative (0), if < 1% of cells displayed positive nuclear staining. Those cases with > 1% of tumor cells showing nuclear staining for USP22 were classified as positive and graded as 1+ (1 - 5%), 2+ (5 - 24%), and 3+ (> 25% of the cells stained positive) as previously described 13, 28. IHC images were acquired using an Aperio AT2 slide scanner.

For multiplex immunofluorescence staining (mIF) assay**,** slides were baked at 60°C for 1 h, dewaxed in two 5 min rounds of xylene substitute, and rehydrated through a graded ethanol series: two rounds each of 100% and 95% reagent alcohol for 5 min, followed by 70% RA for 5 min and 50% RA for 3 min. Rehydration was completed with three 3-min washes in MilliQ water. Antigen retrieval was performed using 1X citrate buffer for 20 min, followed by cooling at room temperature for 30 min, two TBS washes, and one TBST wash (5 min each on an orbital shaker). Slides were blocked with 6% donkey serum and 0.3M glycine in TBST for 1 h, then incubated overnight at 4°C with primary antibodies in blocking buffer. Controls received blocking buffer without primary antibody. Primary antibodies and dilutions included: 5 µg/mL of CD4 (ThermoFisher Scientific, Cat#: 14-9766-82); 1:250 of CD8 (Abcam, Cat#: ab237723); 1:100 of CD68 or F4.80 (ThermoFisher Scientific, Cat#: MA5-16674), 10 µg/mL of CD206 (R&D Systems, Cat#: AF2535), 1:100 of Foxp3 (ThermoFisher Scientific, Cat#: PA5-142112), 1:500 of Ki67 (ThermoFisher Scientific, Cat#: 14-5698-82); 1:50 of PD-L1 (Rockland Immunochemicals, Cat#: 600-101-GU8), 1:50 of SFTPC (pulmonary-associated surfactant protein C, ThermoFisher Scientific, Cat#: BS-10067R), and 1:200 of USP22 (Abcam, Cat#: ab195289). After primary incubation, slides were washed three times in TBST, then incubated for 1 h at room temperature with a 1:500 dilution of secondary antibodies. Following secondary incubation, slides were stained with DAPI (1:4000 in TBS) for 8 min, washed (2× TBST, 1× TBS), mounted using Fluoro-Gel with Tris, and coverslipped. Fluorescent slides were imaged on a Zeiss Axioscan 7 at 20X magnification (0.344 μm/pixel) using consistent exposure times. Slides were mounted with VECTASHIELD mounting media containing DAPI or propidium iodide (PI). IHC slides were scanned using the Aperio AT2. MIF panels were developed by the City of Hope Immuno-Oncology Core and Visikol (https://visikol.com/) 29. Cell types, densities, and associations were analyzed using QuPath v0.5.1 with a machine learning cell classifier 30.

### RNA-sequencing and Visium 10x spatial transcriptomic profiling

Bulk RNA sequencing (RNA-seq) was performed on total RNA extracted from the tumor cores of six KP and six KPU- tumors (∼1 × 10^6^ cells). Differential expression between KPU- and KP samples was assessed using the Bioconductor package edgeR v3.32.1 31 with a design matrix and quasi-likelihood F-tests. Differentially expressed gene signatures and enriched pathways were identified using gene-set enrichment analysis (GSEA) and DAVID 6.7, as previously reported 13, 28. Spatial transcriptomic profiling was performed following the manufacturer’s instructions (10x Genomics). Tissue sections were H&E-stained, reviewed by pathology, and prepared for test sequencing using the Visium Tissue Section Test Slide (10x Genomics, Cat. PN-2000460). Fragment size (> 200 nucleotides) was assessed with the 4200 TapeStation System (Agilent Technologies). FFPE sections (5 μm) were rehydrated in a 42 °C water bath, mounted on Visium Spatial Gene Expression Slides (10x Genomics), dried at 42 °C for 3 h in a desiccation chamber, and deparaffinized with Deparaffinization Solution (Qiagen, Cat. 19093) at 60 °C for 2 h. H&E staining was performed per Visium technical guidelines, and images were acquired using a Zeiss Axioscan2 microscope (10x objective). Decrosslinking followed the 10x Genomics CG000407 protocol, after which sections were hybridized with the Visium Mouse Transcriptome Probe Set v1.0 (20,551 genes, 20,873 probes). Libraries were prepared with the Dual Index Kit TS, Set A (10x Genomics, Cat. PN-1000251) per the Visium CytAssist Spatial Gene Expression for FFPE-Tissue Preparation Guide (CG000495) and sequenced on an Illumina platform (paired-end, 2 x 100 cycles). Sequencing data were processed with Space Ranger v2.0.1 (10x Genomics) for alignment to the mm10-2020-A transcriptome, tissue and fiducial detection, UMI counting, and generation of spatial gene expression matrices. Downstream analysis used Seurat v5.0.0 32 with sctransform v0.4.1 33 for normalization and harmony v1.2.3 34 for integration.

### Spatial analysis of 10x Visium and mIF image data

Spatial transcriptomic data generated using the 10x Genomics Visium platform were processed using standard quality-control and normalization workflows in Seurat/Scanpy. Tissue spots with low transcript counts or high mitochondrial RNA content were excluded prior to downstream analysis. Cell type-associated signatures were inferred using curated marker genes, and Cd8a-high regions were identified based on normalized Cd8a (CD8 T cell) expression levels across Visium spots.

To evaluate spatial relationships between Cd8a-enriched regions and Mrc1 (CD206, M2 macrophage) expression, Euclidean distances were calculated from each Visium spot to the nearest Mrc1-positive spot centroid. Spatial enrichment curves were generated by comparing the observed probability of Cd8a-high spot occurrence across increasing distances from Mrc1-positive regions against a randomized baseline model generated by spatial permutation of spot labels while preserving tissue geometry. Probability values >1 indicate enrichment relative to baseline expectation, whereas values < 1 indicate depletion. Curves were smoothed using a locally weighted regression approach for visualization.

MIF images were analyzed to quantify spatial relationships between cell populations in KP and KPU- tumors. Cell phenotypes were identified by marker-based segmentation and classification, followed by extraction of single-cell spatial coordinates. Spatial clustering was assessed using Ripley’s L function across increasing radial distances (0 - 150 μm), as reported previously 35. Curves above the randomized baseline (dotted line) indicate spatial clustering, with higher values reflecting stronger aggregation. Individual curves represent independent regions or samples within each group. Spatial interaction enrichment analysis was performed on mIF images using single-cell spatial coordinates and phenotype annotations using Squidpy 36. Cells were classified into indicated populations based on expression markers of CD8/CD4/Foxp3/F4/80/CD206 (CD4+/Foxp3+ for Tregs, F4/80+/CD206+ for M2 macrophages) and pairwise spatial interactions between cell types were quantified within a defined neighborhood radius. Observed interaction frequencies were compared against randomized spatial permutations to generate enrichment z-scores, where positive values indicate spatial colocalization/enrichment and negative values indicate spatial avoidance. The resulting interaction matrix was visualized as a heatmap summarizing pairwise spatial relationships among immune and tumor cell populations.

### Micro-CT imaging

Mice were anesthetized by inhalation of isoflurane and scanned using the Quantum GX2 micro-CT imaging system (Perkin Elmer). To evaluate the impact of Usp22-KO on tumor growth in vivo, changes in tumor volume were measured over 4 weeks through serial CT scans. Serial lung images were reconstructed, and tumor volumes were subsequently analyzed using Analyze-15 (AnalyzeDirect, Overland Park, Kansas, USA).

### Enzyme-linked immunosorbent assay

Mouse transforming growth factor beta 1 (TGF-β1), secreted protein acidic and rich in cysteine (SPARC, also known as Osteonectin), and monocyte chemoattractant protein-1/ C-C motif chemokine ligand 2 (MCP-1/CCL2) were quantified from 200 μg of tumor protein lysates using enzyme-linked immunosorbent assay (ELISA) kits purchased from MyBioSource (San Diego, CA) according to the manufacturer’s instructions.

### Macrophage migration and differentiation and polarization assays

The murine macrophage-like cell line RAW 264.7 was cultured in Dulbecco’s modified Eagle Medium (DMEM) supplemented with 10% fetal bovine serum (FBS). To assess the effect of SPARC on RAW 264.7 cell migration, a Transwell Boyden chamber assay was performed. RAW 264.7 cells were seeded into the upper chamber in serum-free medium. Recombinant mouse SPARC (500 ng/mL) was added to the lower chamber as a chemoattractant. The cells were incubated for 24 h at 37 °C After incubation, cells that migrated to the lower side of the membrane were fixed, stained, and counted.

For macrophage polarization studies, RAW 264.7 cells were treated to induce differentiation into M1-like or M2-like phenotypes. Cells were incubated for 24 h with either control medium, medium containing lipopolysaccharide (LPS; 50 ng/mL) plus interferon-gamma (IFN-γ; 10 ng/mL) to induce M1-like polarization, or medium containing transforming growth factor-beta 1 (TGF-β1, 5 ng/mL) to induce M2-like polarization. Following polarization, cells were stained using antibodies specific to macrophage markers: CD86 (M1 marker) and CD206 (M2 marker). Double staining was performed, and the proportion of cells positive for each marker was quantified.

### Determining the effect of Usp22-KO on anti-PD-L1 therapy in mouse KP-lung cancer

KP (16 mice) and KPU- (16 mice) lung cancers were induced as described above. Mice were imaged twice weekly by bioluminescence imaging to monitor primary lung cancer initiation and growth. Treatments were initiated on day 50 - 55 after Ad-Cre induction. Cancer-bearing mice were injected intraperitoneally 2 times/week with 10 mg/kg of anti-mouse PD-L1 antibody (10F.9G2, Bio X Cell) or isotype-matched control IgG (Ctrl Ab, LTF-2, Bio X Cell) 37. Tumor growth was monitored twice a week by bioluminescence imaging. Body weight was recorded every five days. Animals were euthanized three days after the fourth injection of anti-PD-L1 antibody (anti-PD-L1 Ab). Tumors were collected and processed as described above. Half (8/16) of the mice were maintained for metastasis and survival analysis.

### Murine KP and KPU- cancer cells and conditioned medium generation

Murine primary KP and KPU- cancers were generated as described above. After tumor formation, lung tumors were harvested under sterile conditions, mechanically dissociated, and cultured to establish stable murine KP and KPU- lung cancer cell lines. Cells were maintained in RPMI supplemented with 10% FBS and passaged every 3 days. For conditioned medium (CM) collection, equal numbers of KP and KPU- cells (passages 8 - 10) were seeded in 10-cm plates in RPMI with 10% FBS overnight to reach 80 - 90% confluence. Medium was then replaced with RPMI containing 2.5% FBS and cultured for 48 h. Supernatants were collected, centrifuged to remove debris and dead cells, normalized by cell number, and adjusted to 1 mL CM per 1 × 10⁶ cells, designated KP-CM and KPU-CM, and used for subsequent experiments.

### Subcutaneous KP and KPU- cancers treated with anti-PD-1 antibody

For syngeneic cancer studies, KP and KPU- cells (passages 8 - 10) were harvested during the logarithmic growth phase, washed twice with sterile PBS, and resuspended in a 3:1 mixture of RPMI-1640 and Matrigel. A total of 5 × 10⁶ cells in 100 μL were injected subcutaneously into the flanks of 6-8-week-old C57BL/6J mice (equal numbers of males and females). Cancer growth was monitored twice weekly using digital calipers, and tumor volume was calculated as V = (length × width²) / 2.

When the tumor volume reached ~100 mm³, mice were randomized into two groups (n = 7 per group) and treated by intraperitoneal injection with 200 μg Ctrl Ab or anti-PD-1 antibody (anti-PD-1 Ab, Bio X Cell, clone: J43) every 3 days for a total of five doses. After treatment, mice were euthanized and tumors were excised, weighed, and photographed. Tumor sections were subjected to IHC staining for USP22, F4.80, CD206, and SPARC as described previously 27. Stained sections were scanned and imaged by light microscopy. Positive cells were quantified in at least five randomly selected high-power fields per tumor using image analysis software, and the percentage of positive cells was calculated relative to the total number of tumor cells.

### Statistical analysis

Pairwise statistical comparisons were performed using Student’s unpaired 2-sided *t* test. Cancer differentiation (well, moderate, poor) was compared between KP and KPU- cancers using Chi-square (χ²) test of independence. The log-rank test was used to compare survival curves. GraphPad PRISM 8 (GraphPad Software, LLC, San Diego, CA, USA) and R (The R Foundation, Vienna, Austria) was used for data processing, statistical analysis, and result visualization. R version 4 was utilized for the statistical and bioinformatics analysis of RNA-seq data. The R packages used for all analyses described in this manuscript were obtained from Bioconductor and the Comprehensive R Archive Network (CRAN) repositories. On graphs, error bars represent the range of standard deviation (SD) of the mean, as indicated in legends. For all figures, *P* < 0.05 was considered statistically significant; *P < 0.05; **P < 0.01; ***P < 0.001.

## Results

### Usp22-KO significantly altered differentiation, suppressed KRAS/p53-driven mouse lung cancer growth, and prolonged survival in tumor-bearing mice

**Figure 1:**
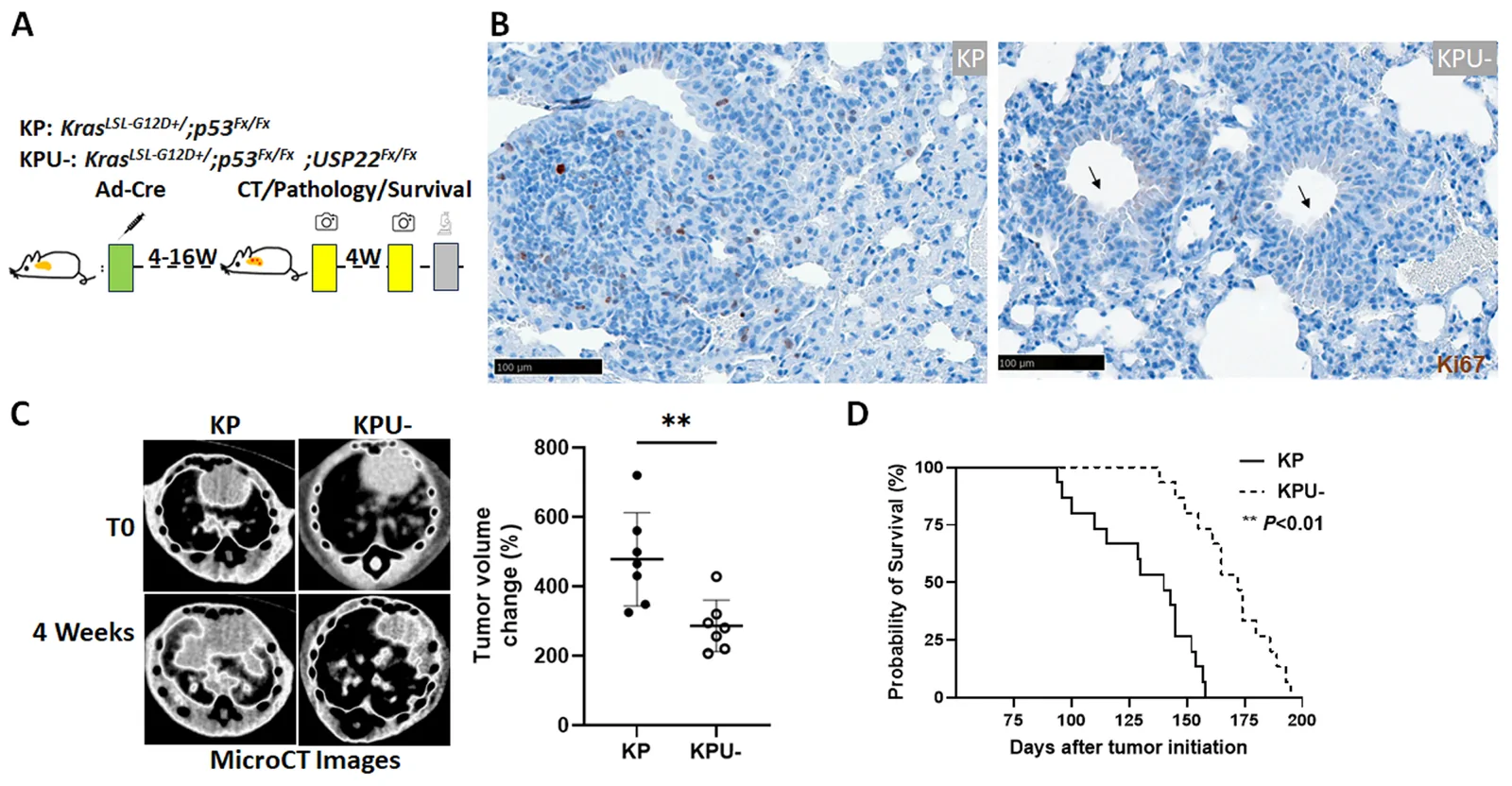
** Usp22-KO significantly altered differentiation, suppressed KRAS/p53-driven mouse (KP) lung cancer growth, and prolonged survival in cancer-bearing mice.** (**A**) The animal experiment protocol. (**B**) IHC of Ki67 (scale bar: 100 µm, 100x magnification) in KP and KPU- mouse lung tissues at 8 weeks post-administration of Ad-Cre, arrows point to adenoma lesions in KPU- mouse lung tissues. (**C**) Micro-CT images of representative KP and KPU- lung cancers at T0 and 4 weeks later (left panel), and quantitative analysis of cancer volume increases over 4 weeks in KPU- cancers compared with parental KP cancers (n = 7). (**D**) Kaplan-Meier survival analysis of KP and KPU- cancer-bearing mice (n = 15), *P < 0.05, **P < 0.01, KP vs. KPU-.

Previous studies from our group have demonstrated that Usp22-KO significantly inhibits cancer stemness and *in vivo* growth of human KRAS-mutant lung cancer xenografts 13, 14. In the present study, Usp22-KO KP mice were generated to further investigate the role of USP22 in carcinogenesis and immune response in KRAS-driven mouse lung cancer. The *in vivo* experimental procedure for KP-induced mouse lung cancer study is outlined in **Figure 1A**. Six mouse lungs from each group were dissected and pathologically analyzed at 4 to 16 weeks following the initiation of lung carcinogenesis induced by Ad-Cre administration. We first examined the temporal expression of USP22 during KP-driven lung cancer development. IHC analysis at 4 weeks post-Ad-Cre administration revealed that USP22 protein was heterogeneously and weakly expressed in KP-induced carcinogenesis (data not shown). However, by 8 weeks post-initiation, IHC analysis showed clear USP22 protein expression in KP lung cancer tissues, whereas USP22 protein was undetectable in KPU- tumors, confirming the effective knockout of USP22 (**Figure S1**). Notably, consistent with the more advanced stage of cancer development, KP cancer lesions exhibited stronger staining for the proliferation marker Ki67 than KPU- lesions at the same post-initiation time point (**Figure 1B**). H&E staining and pathological analysis were used to characterize cancer lesion stages. Based on morphological features, lesions >150 µm in diameter were classified as adenomas, adenocarcinoma in situ (AIS), or invasive adenocarcinomas, and the proportion of each type was quantified. The analysis showed that AIS and invasive adenocarcinomas were prevalent in KP lungs, whereas most lesions in KPU- lungs were adenomas (**Figure S2A,** *P* < 0.01). Consistent with these findings, at later time points (16 - 20 weeks post-initiation), well-to-moderately differentiated adenocarcinomas were still observed in KPU- lungs, whereas poorly differentiated adenocarcinomas were predominantly found in KP lungs; the difference was statistically significant (**Figure S2B**, *P* < 0.01). Collectively, these results indicate that Usp22-KO slows lung tumor progression and is associated with improved tumor differentiation.

We further investigated the effect of Usp22-KO on *in vivo* KP lung cancer growth and overall prognosis during cancer progression. Dual IHC staining for USP22 and Ki67 was performed on lung cancer tissues harvested at 16-20 weeks post-initiation of carcinogenesis. Quantitative analysis revealed a significantly lower percentage of Ki67-positive tumor cells in KPU- tissues compared to KP tissues, indicating reduced proliferative activity in the absence of USP22 (**Figure S2C**). Consistent with this observation, gross examination showed that both the wet weights of whole lungs (**Figure S3A-B;** at 10-, 13-weeks post cancer initiation**)** and cancer areas identified by H&E staining (**Figure S3C**) were markedly smaller in KPU- mice than in KP mice at the same timepoints (12 weeks) after cancer initiation. To further assess the impact of Usp22-KO on tumor growth, comparably sized tumors from KP and KPU- mice were selected based on CT imaging. Serial CT scans were then conducted to monitor tumor volume changes over a 4-week period. The analysis demonstrated that tumor volume increase in KPU-mice was significantly slower than in KP mice, indicating tumor growth in KPU- mice was significantly slower than in KP mice (**Figure 1C**). To evaluate the effect of Usp22-KO on survival, we performed Kaplan-Meier survival analysis. Mice bearing KPU- lung tumors showed a significantly extended survival compared to KP mice (KP: mean ± SD = 131 ± 23 days; KPU-: mean ± SD = 169 ± 18 days; n = 15, *P* < 0.01; **Figure 1D**). In summary, while USP22 knockout did not completely inhibit KP-driven lung tumor formation, it significantly suppressed tumor growth, improved tumor differentiation, and prolonged survival in affected mice.

### Usp22-KO significantly modulated multiple cancer-associated signaling pathways

**Figure 2:**
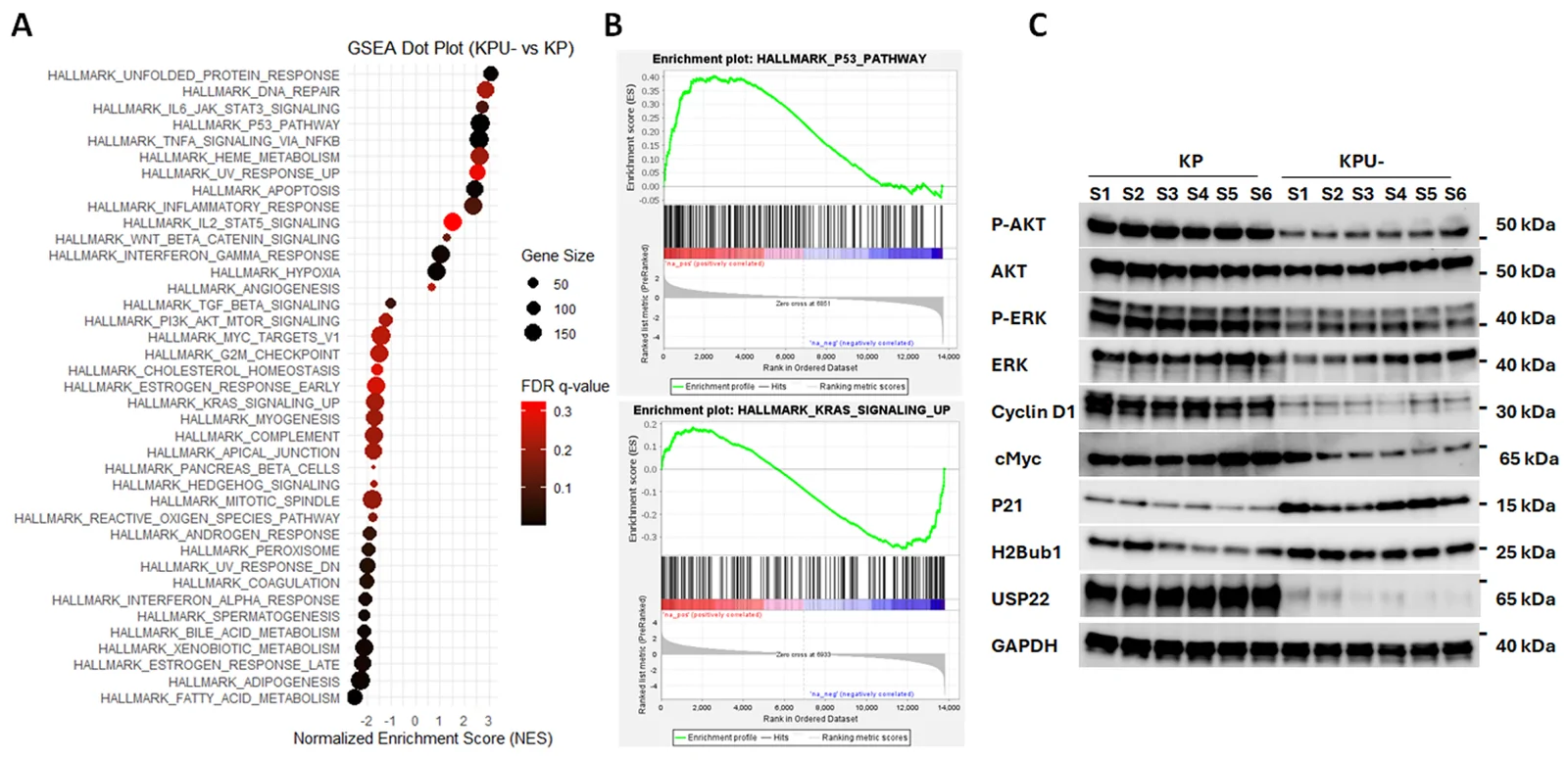
** Usp22-KO modulated cancer-related signaling pathways in KP mouse lung cancers.** (**A**) Modulated signaling pathways shown by gene set enrichment analysis. (**B**) Enrichment plots for enhanced P53 (upper panel) and attenuated KRAS signaling pathways. (**C**) Western blot analysis of phosphorylated AKT, ERK, p21, cyclin D1, H2Bub1, USP22 in KP and KPU- lung cancers.

One of the critical functions of USP22 in cancer is its role in transcriptional regulation through modulation of H2Bub1 10, a mechanism also observed in human lung cancer cells 13, 28. The biological and molecular functions of USP22 in KP lung cancer were investigated through RNA-seq by profiling global gene expression changes following Usp22-KO. Usp22-KO resulted in significant alterations in the expression of key cancer-related genes. GSEA of hallmark ontology signaling pathways identified multiple pathways impacted by Usp22-KO in KP lung cancer. Processes such as angiogenesis, G2M checkpoint, PI3K-AKT-mTOR, KRAS signaling, and c-Myc signaling were significantly downregulated in KPU- lung cancers (**Figure 2A**). Conversely, signaling pathways related to DNA repair, UV response, apoptosis, and inflammatory, interferon-gamma, and TNF-alpha were upregulated (**Figure 2A**). Notably, KRAS signaling was downregulated, while the p53 signaling pathway was upregulated in KPU- lung cancers compared to parental KP cancers, which suggests that Usp22-KO partially suppresses the oncogenic activity of KRAS while compensating for the loss of p53 tumor suppressor function (**Figure 2B**). Unsupervised heatmaps revealed the differentially expressed genes associated with KRAS and p53 signaling pathways in KP and KPU- lung cancer samples (n = 4, **Figure S4**). Western blot analysis corroborated these findings, showing a moderate increase in H2Bub1 levels and significant suppression of the AKT and ERK signaling pathways in KPU- lung cancers (**Figure 2C**). These results provide further evidence of KRAS pathway inhibition in the absence of USP22. Usp22-KO also influenced the expression of key genes involved in cell cycle progression and proliferation. c-Myc, a well-known target of USP22, plays a crucial role in regulating cell growth and metabolism, was downregulated, and Cyclin D1, which promotes G1/S phase transition, was downregulated, while p21, a cyclin-dependent kinase inhibitor, was upregulated (**Figure 2C**). In summary, these data demonstrate that Usp22-KO profoundly impacts multiple pathways critical for KRAS-mutant lung cancer progression.

### Usp22-KO significantly modified immune landscape highlighted by increased T cell infiltration and decreased macrophages in the TME of KP lung cancer

**Figure 3:**
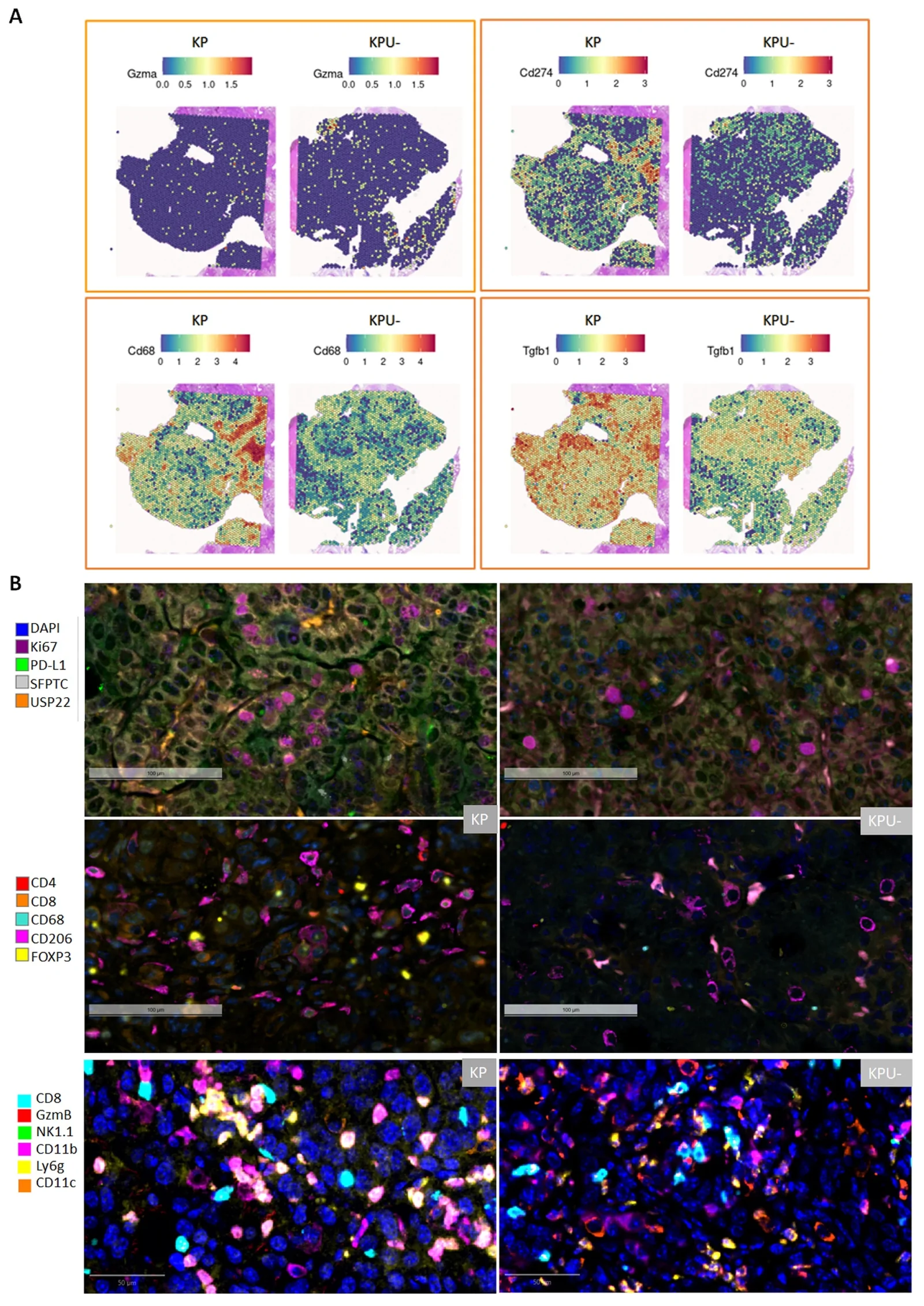

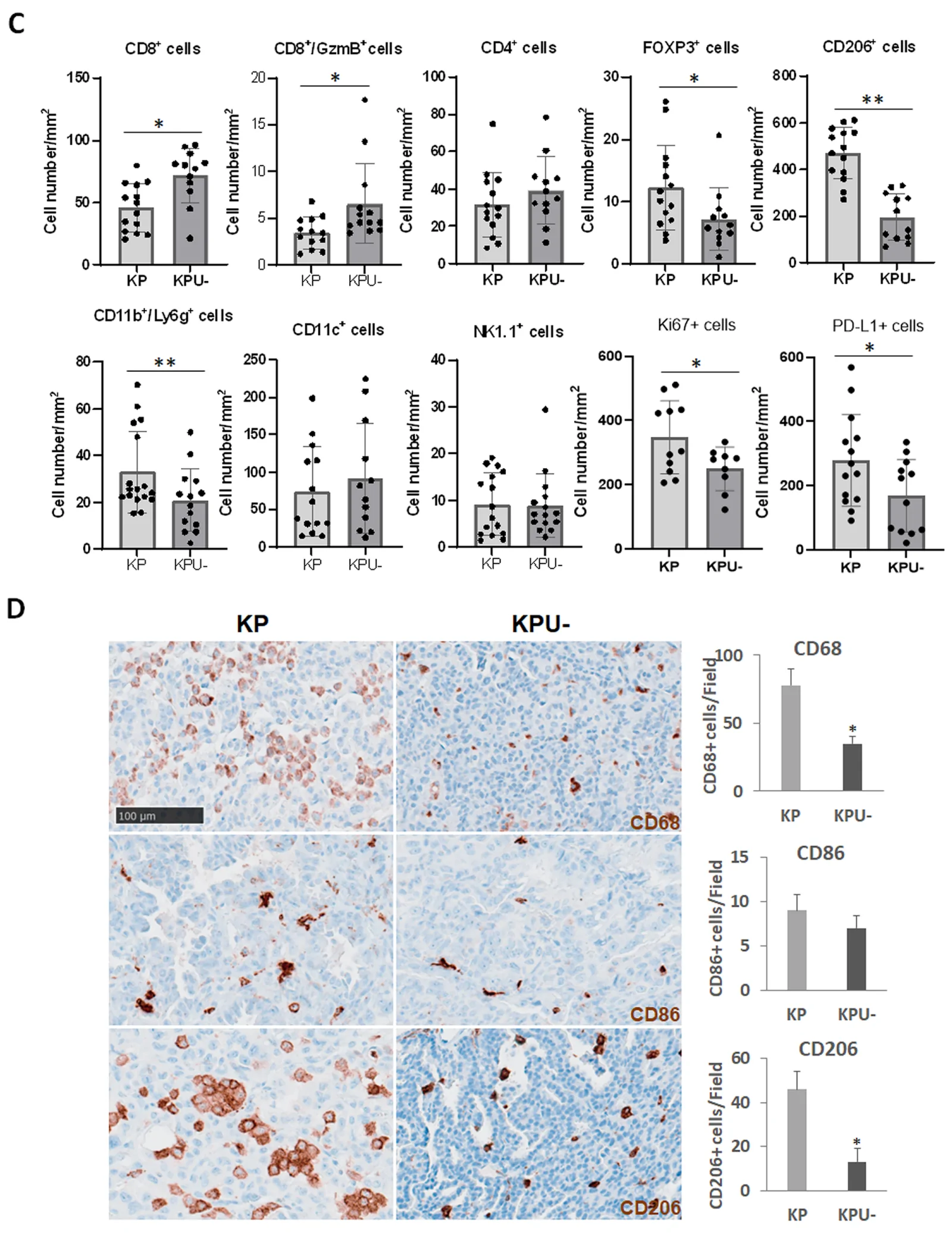
** Usp22-KO enhanced cytotoxic T cells while decreased M2 macrophages and MDSCs in mouse KP lung cancer.** (**A**) Spatial transcriptomic plots and Violin plots of expression levels of GZMA (granzyme A, cytotoxic biomarker), CD274 (PD-L1), CD68 (pan-macrophage marker), TGF-β1 in a representative KP and KPU- cancer regions. (**B**) mIF images of Ki67/PD-L1/SFTPC/USP22 (upper panel) and CD4/CD8/CD68/CD206/Foxp3 (middle panel), and GzmB (granzyme B, cytotoxic cell marker), NK1.1 (NK cell marker), CD11b/Ly6g (MDSC marker), CD11c (dendritic cell marker) (lower panel, scale bar: 50 µm) in representative KP (left panel) and KPU- (right panel) cancers. (**C**) Semi-quantitative analysis of mIF images in KP and KPU- lung cancers (n = 9 - 14 cancer nodules with surface area > 2 mm^2^ from 7 - 10 mice). (**D**) IHC staining (scale bar: 100 µm, 200x magnification) of CD68**,** CD86 (M1 macrophage marker) and CD206 (M2 macrophage marker) in KP and KPU- lung cancers and quantitative analysis of positively stained cells per field in KP vs. KPU- lung cancer; n = 7; *P < 0.05, **P < 0.01.

To explore the mechanisms by which Usp22-KO affects TME and immune infiltration further, we performed spatial transcriptomics. Spatial transcriptomics revealed significant differences in the TME of KP and KPU- lung cancers. Compared to KP samples, KPU- samples exhibited increased areas/cells expressing active cytotoxic T cell biomarkers granzyme A (GzmA) 38, along with reduced expression of immunosuppressive markers such as CD274 (PD-L1) and CD68 (macrophage marker) (**Figure 3A**). Notably, TGF-β1, a cytokine that plays a key role in suppressing anticancer immunity 39, was significantly reduced in Usp22-KO tumors (**Figure 3A**). Spatial transcriptomics also revealed a moderate increase in regions expressing CD3 and CD8, while regions expressing CD4 and Foxp3 showed a moderate decrease (**Figure S5**). These findings suggest that Usp22-KO alters immune infiltration in the TME of KP lung cancers. To further characterize immune infiltration, mIF was performed. Representative mIF images of KP and KPU- lung cancers showed the changes in Ki67, PD-L1 and CD8/CD4/Foxp3 stains (**Figure 3B** & **Figure S6**). Quantitative image analysis revealed that Usp22-KO significantly increased the total number of and granzyme B+ (GzmB, cytotoxic cell) CD8+ T cells, particularly reducing Foxp3+ CD4 Tregs, which are known to suppress antitumor immunity (**Figure 3C**). Of note, immunosuppressive myeloid-derived suppressor cells (MDSCs), identified as CD11b+/Ly6G+ double-positive cells, were significantly decreased in KPU- tumors, consistent with a previous report of reduced MDSCs in Usp22-KO pancreatic cancers 40. Unlike previous findings showing increased natural killer (NK) cells in Usp22-KO pancreatic cancers 40, NK cells, defined by NK1.1 staining, were much less abundant than T cells but were not altered in KPU- tumors compared with KP tumors; CD11c+ dendritic cells were also unchanged in KPU- tumors compared with KP tumors. (**Figure 3B-C**). Additionally, mIF analysis confirmed that PD-L1 expression was markedly reduced in Usp22-KO tumors, supporting the hypothesis that Usp22-KO enhances antitumor immune responses in KP lung cancers. Notably, mIF analysis showed a significant decrease in CD206+ M2 macrophage infiltration in KPU- tumors (**Figure 3B-C**), which potently suppress CD8+ T cell function leading to reduced T cell activity, cytokine production, and tumor-killing capacity 41, 42. A reduction in Ki67-positive cancer cells in KPU- compared to KP tumors was also observed (**Figure 3B-C**), indicating suppressed tumor growth upon Usp22-KO. To further investigate if M1 (pro-inflammatory) macrophages were affected in comparison with M2 (anti-inflammatory, immunosuppressive) macrophages by IHC analysis in KP and KPU- lung tumors. We observed that while total macrophages (CD68-positive) and M2 macrophages (CD206-positive) were significantly reduced in Usp22-KO tumors, M1 macrophages (CD86-positive) were extremely low in KP and KPU- lung cancers, and the abundance of M1 remained unchanged (**Figure 3D**). These findings suggest that Usp22-KO significantly alters the immune landscape of KP lung cancers by reducing PD-L1 expression in cancer cells and suppressing M2 macrophage infiltration, Usp22-KO promoting antitumor immunity.

### Spatial interaction analysis of CD8+ T cells and M2 macrophages

**Figure 4:**
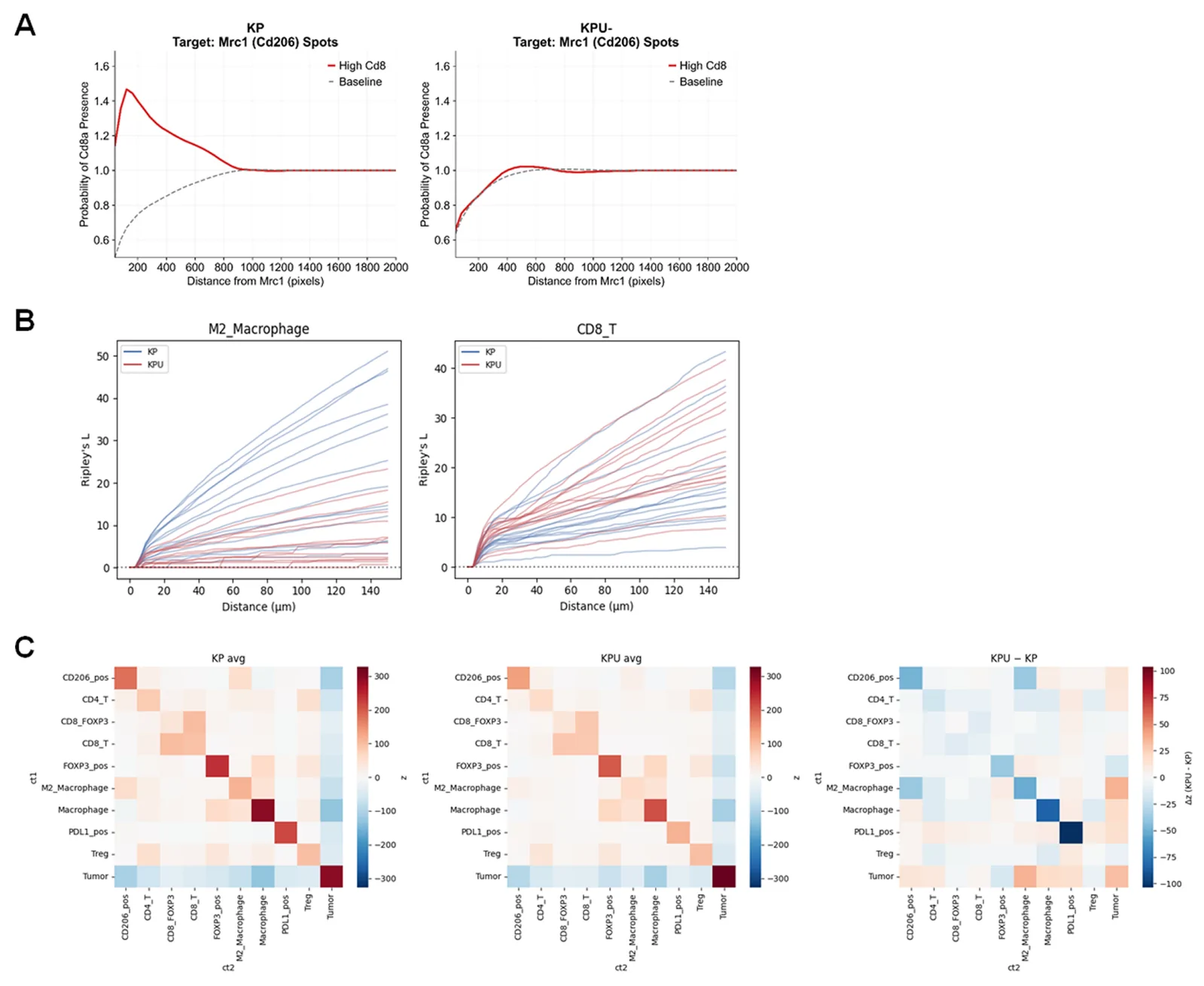
** Spatial analysis of interactions between CD8+ T cells and M2 macrophages in KP and KPU- lung cancers**. (**A**) Spatial association of Cd8a-high spots (CD8+ T cells) relative to Mrc1-positive regions (CD206+ M2 macrophages) in 10x Visium data. The red line indicates the observed probability of Cd8a-high spot occurrence as a function of distance from Mrc1-positive spots, and the dashed gray line indicates the randomized expectation. Cd8a-high spots are enriched near Mrc1-positive regions in KP cancers (left panel) but show weaker spatial association in KPU- cancers (right panel). Distances are shown in pixel units. (**B**) Spatial clustering analysis of cell populations in mIF images using Ripley’s L function. Ripley’s L curves showing the spatial organization of M2 macrophages (left panel) and CD8+ T cells (right panel) in KP cancers (blue line, n = 14) and KPU- cancers (red line, n = 14) across increasing distances (μm). Each line represents an individual sample or region of interest. (**C**) Spatial interaction heatmaps of major cell populations. Average spatial interaction scores in KP cancers (left panel) and KPU- cancers (middle panel), with the differential interaction map (KPU- - KP; right panel) showing relative changes in cell-cell associations. Blue indicates stronger interactions in KP cancers, and red indicates stronger interactions in KPU- cancers.

Spatial analysis of Visium spatial transcriptomics demonstrated a strong enrichment of Cd8a-high (encoding the CD8 α chain protein, a marker for CD8+ T cells) regions proximal to Mrc1 (macrophage mannose receptor encoding Mrc1/CD206 protein, a marker for M2 macrophages)-positive areas in the KP tumors, with peak enrichment occurring within approximately 100 - 250 pixels from Mrc1-positive spots (**Figure 4A** left-panel). This enrichment gradually declined with increasing distance and approached baseline levels beyond ~1,000 pixels, indicating localized immune aggregation near Mrc1-expressing regions. In contrast, the KPU- sample showed only modest and transient Cd8a enrichment near Mrc1-positive regions, with probabilities remaining close to baseline across most distances (**Figure 4A,** right-panel). These findings suggest heterogeneous spatial coupling between immune infiltration and Mrc1 expression across samples. The red line indicates the observed probability of Cd8a-high spot occurrence as a function of distance from Mrc1-positive spots, while the dashed gray line represents the randomized baseline expectation.

Spatial interaction analysis of mIF images also revealed distinct immune architectures between KP and KPU- tumors. In **Figure 4B** left panel, KP (blue) samples exhibited markedly stronger spatial clustering of M2 macrophage cells, with several curves reaching substantially higher Ripley’s L values than KPU- (red) samples. In contrast, KPU- tumors showed comparatively weaker and more spatially restricted clustering of M2 macrophage cells. In **Figure 4B** right panel, both KP and KPU- samples demonstrated heterogeneous spatial clustering patterns of CD8+ T cells across samples. However, KPU- tumors generally exhibited moderately greater spatial organization of CD8+ T cells, with more red curves trending above the blue curves across increasing distances, indicating enhanced cellular aggregation of CD8+ T cells relative to KP tumors despite substantial inter-sample variability. Interaction heatmaps showed that KP and KPU- tumors shared broadly similar overall spatial organization of immune cells, but several macrophage-centered interactions were consistently stronger in KP tumors. In KP tumors (**Figure 4C**, left panel), M2 macrophages and macrophages displayed increased spatial associations with CD8+ T cells, Treg cells, and PD-L1+ populations compared with KPU- tumors (**Figure 4C**, middle panel), where these interactions were reduced. Consistently, the differential heatmap (**Figure 4C**, right panel) highlighted decreased M2 macrophage-CD8+ T-cell and macrophage-PD-L1+ interactions in KPU- relative to KP, together with weaker Treg-associated interactions. Overall, these findings suggest that USP22 loss does not drastically alter the global neighborhood architecture but selectively attenuates macrophage-centered and immunoregulatory cellular organization. KP tumors therefore retain more tightly organized immune niches enriched for macrophage and suppressive immune cell interactions, whereas these associations are partially disrupted in KPU- tumors.

### Usp22-KO significantly sensitized KP lung cancer to anti-PD-L1 therapy

**Figure 5:**
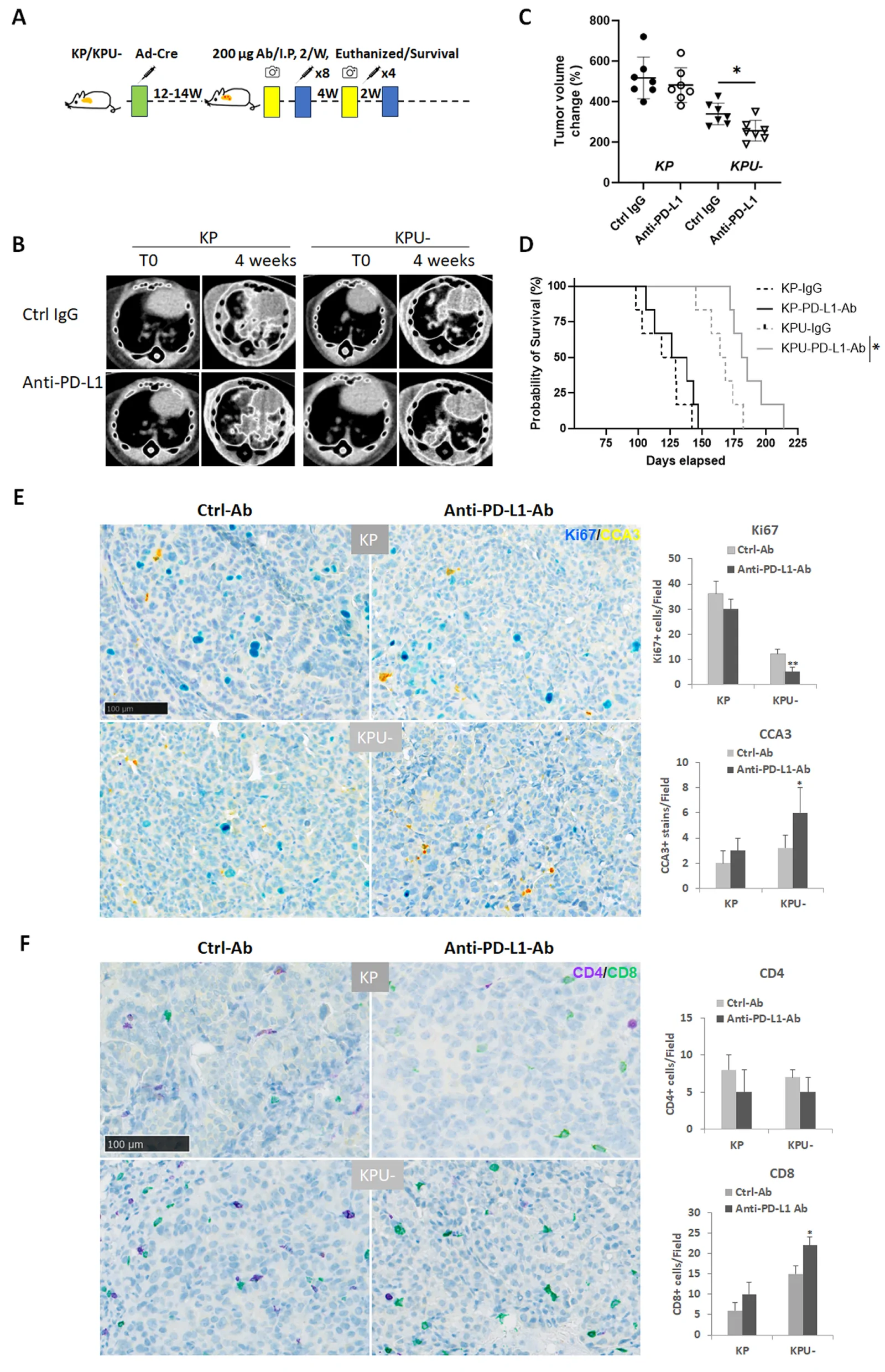
**Usp22-KO significantly sensitized KP lung cancer to anti-PD-L1 antibody.** (**A**) Treatment procedure of the animal experiment. (**B**) Micro-CT images (left panel) of a representative KP and KPU- lung cancer pre- (T0) and 4 weeks post-treatment of control IgG (Ctrl Ab) or anti-PD-L1 antibody (anti-PD-L1 Ab). (**C**) Semi-quantitative analysis of cancer volume changes over 4 weeks in KP and KPU- mice treated with Ctrl Ab or anti-PD-L1 Ab (n = 7 or 8 per group;), data are mean per mouse (large symbols) ± SD; small symbols, individual tumors (n = 8). (**D**) Kaplan-Meier survival analysis of KP and KPU- mice treated with Ctrl Ab or anti-PD-L1 Ab for 6 weeks (n = 7 per group). (**E**) IHC of Ki67 (blue, scale bar: 100 μm; 200× magnification) and apoptosis marker cleaved caspase-3 (CCA3, yellow) in representative KP and KPU- lung cancer tissues (left panel), semi-quantitative analysis of Ki67 and CCA3 (right panel). (**F**) IHC of CD4 (purple) and CD8 (green) in KP and KPU- lung cancer tissues treated with Ctrl Ab and anti-PD-L1 Ab (left panel) and semi-quantitative analysis of CD4- and CD8- positive T cells (right panel) in KP and KPU- lung cancer tissues, *P < 0.05, **P < 0.01, Ctrl Ab vs. anti-PD-L1 Ab.

Since Usp22-KO significantly altered immune cell infiltration, we hypothesized that USP22 deficiency might sensitize KPU- lung tumors to anti-PD-L1 ICI therapy. To test this hypothesis, we evaluated the impact of Usp22-KO on anti-PD-L1 antibody treatment in a genetically engineered mouse model of KP lung cancer. As outlined in **Figure 5A**, lung tumors were induced in mice, which were then monitored and treated intraperitoneally with either anti-PD-L1 antibody or Ctrl Ab for 4 weeks. The results demonstrated that Usp22-KO significantly enhanced the therapeutic response to anti-PD-L1 treatment. Representative CT scan images obtained before and after treatment are shown in **Figure 5B.** Three days after the final treatment, mice were euthanized for pathological analysis. Semi-quantitative analysis revealed that tumor volume increase was markedly reduced in KPU- tumors following anti-PD-L1 therapy, whereas KP tumors exhibited minimal or no response to the treatment (**Figure 5C**). In alignment with the data above, the survival benefit of a 6-week anti-PD-L1 antibody treatment was also evaluated in mice bearing KP and KPU- lung cancers. Kaplan-Meier survival analysis revealed that anti-PD-L1 antibody treatment resulted in only a slight, non-significant extension of survival in KP cancer-bearing mice. In contrast, a significant survival benefit was observed in mice bearing KPU- lung cancer (**Figure 5D**). By IHC analysis, we further demonstrated that the enhanced therapeutic efficacy in KPU- tumors was accompanied by increased apoptosis and suppressed tumor growth, as evidenced by IHC staining for cleaved caspase-3 (CCA3) and Ki67, respectively (**Figure 5E**). Moreover, we observed significantly higher infiltration of CD4+ and CD8+ T cells in KPU- cancers treated with anti-PD-L1 antibody compared to KP cancers (**Figure 5F**). This suggests that the increased sensitivity of KPU- tumors to anti-PD-L1 therapy may be, at least in part, mediated by enhanced T cell infiltration. Collectively, these findings demonstrate that Usp22-KO sensitizes KP lung cancers to ICI therapy, leading to more effective tumor suppression in response to anti-PD-L1 treatment.

### Usp22-KO sensitizes subcutaneous KP lung tumors to anti-PD-1 therapy

**Figure 6:**
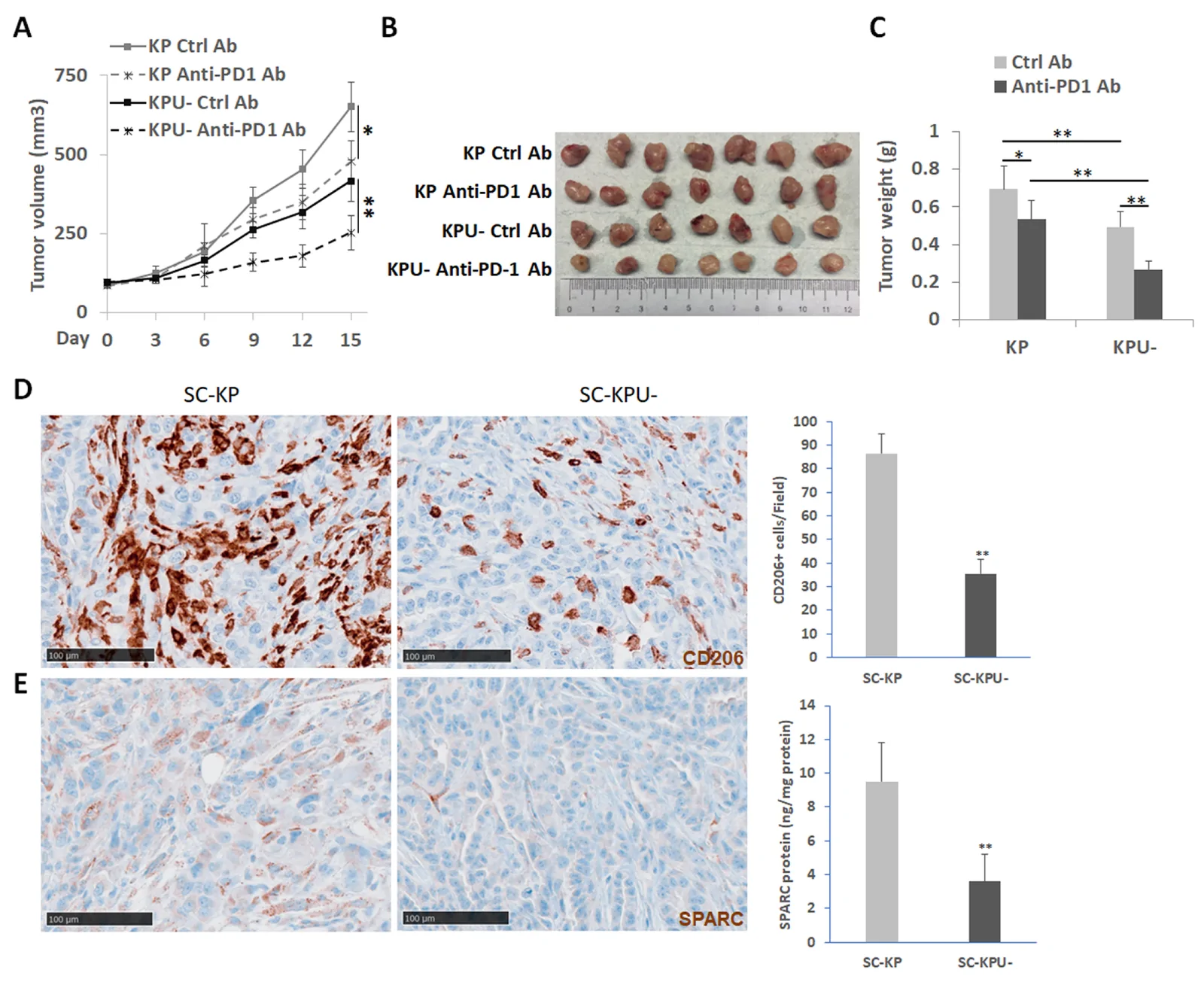
** Usp22-KO significantly enhances the sensitivity of KP lung cancers to anti-PD-1 therapy.** (**A**) Cancer growth curves of subcutaneous KP (SC-KP) and KPU- (SC-KPU-) tumors treated with Ctrl Ab or anti-PD-1 antibody (anti-PD-1 Ab) (n = 7; *P < 0.05, **P < 0.01, anti-PD-1 Ab vs. Ctrl Ab). (**B**) Representative images of tumors from the four groups. (**C**) Quantification of tumor weights of KP/KPU- SC tumors treated with Ctrl Ab or anti-PD-1 Ab (n = 7; **P < 0.01, anti-PD-1 Ab vs. Ctrl Ab and KPU- vs. KP). (**D**) IHC staining (left panel, 200×) and quantification (right panel) of CD206-positive M2 macrophages in SC-KP and SC-KPU- cancers. KPU- tumors exhibited significantly reduced M2 macrophage infiltration compared with KP tumors (n = 7; **P < 0.01, KPU- vs. KP). (**E**) SPARC expression in SC-KP and SC-KPU- cancers. Representative IHC staining (left panel, 200× magnification) and ELISA quantification of SPARC protein levels (right panel) showed significantly reduced SPARC expression in KPU- tumors compared with KP tumors (n = 7; **P < 0.01, KPU- vs. KP).

As shown above, Usp22-KO significantly reduced PD-L1 expression in tumor cells and enhanced tumor sensitivity to anti-PD-L1 ICI therapy. Given the reported synergistic effects between anti-PD-1 and anti-PD-L1 therapies as well as the finding that anti-PD-1 works more efficiently in PD-L1-low advanced lung cancer 43, we next examined whether USP22 deletion also increases tumor responsiveness to anti-PD-1 treatment, since USP22 deletion downregulates PD-L1 in cancers. To further test this, KP and KPU- mouse lung cancer cell-derived subcutaneous tumors were established in syngeneic mice and treated with anti-PD-1 antibodies for 2 weeks. Compared with KP cancers, KPU- cancers displayed slower in vivo growth (**Figure 6A**). Tumor volume measurements showed that anti-PD-1 treatment significantly suppressed the growth of both KP and KPU- tumors compared with those treated with control antibody (**Figure 6A**). Consistently, tumor weights in the anti-PD-1-treated KP and KPU- groups were significantly lower than those in control-treated KPU- tumors (**Figure 6B-C**); however, the magnitude of growth inhibition in KPU- tumors by the treatment was markedly greater than that observed in KP cancers. These results indicate that Usp22-KO enhances cancer sensitivity to anti-PD-1 therapy (**Figure 6B-C**).

In addition, we further analyzed M2 macrophage infiltration in subcutaneous KP and KPU- cancers. Consistent with observations in orthotopic lung cancers induced by Ad-Cre, KPU- cancers contained significantly fewer CD206-positive M2 macrophages than KP cancers (**Figure 6D**). In addition, the expression of SPARC, a multifunctional extracellular matrix (ECM) protein implicated in ECM remodeling, angiogenesis, tumor cell adhesion 44, and macrophage polarization and migration, was examined in these subcutaneous cancers. IHC and ELISA analyses revealed markedly reduced SPARC staining and SPARC protein in KPU- cancers compared with KP cancers (**Figure 6E**).Collectively, these data demonstrate that USP22 loss increased the responsiveness of KP lung tumors to anti-PD-1 therapy and reduced M2 macrophage infiltration and decreased SPARC expression in the TME.

### Usp22-KO significantly decreased SPARC and angiogenesis in KPU- lung cancers

**Figure 7:**
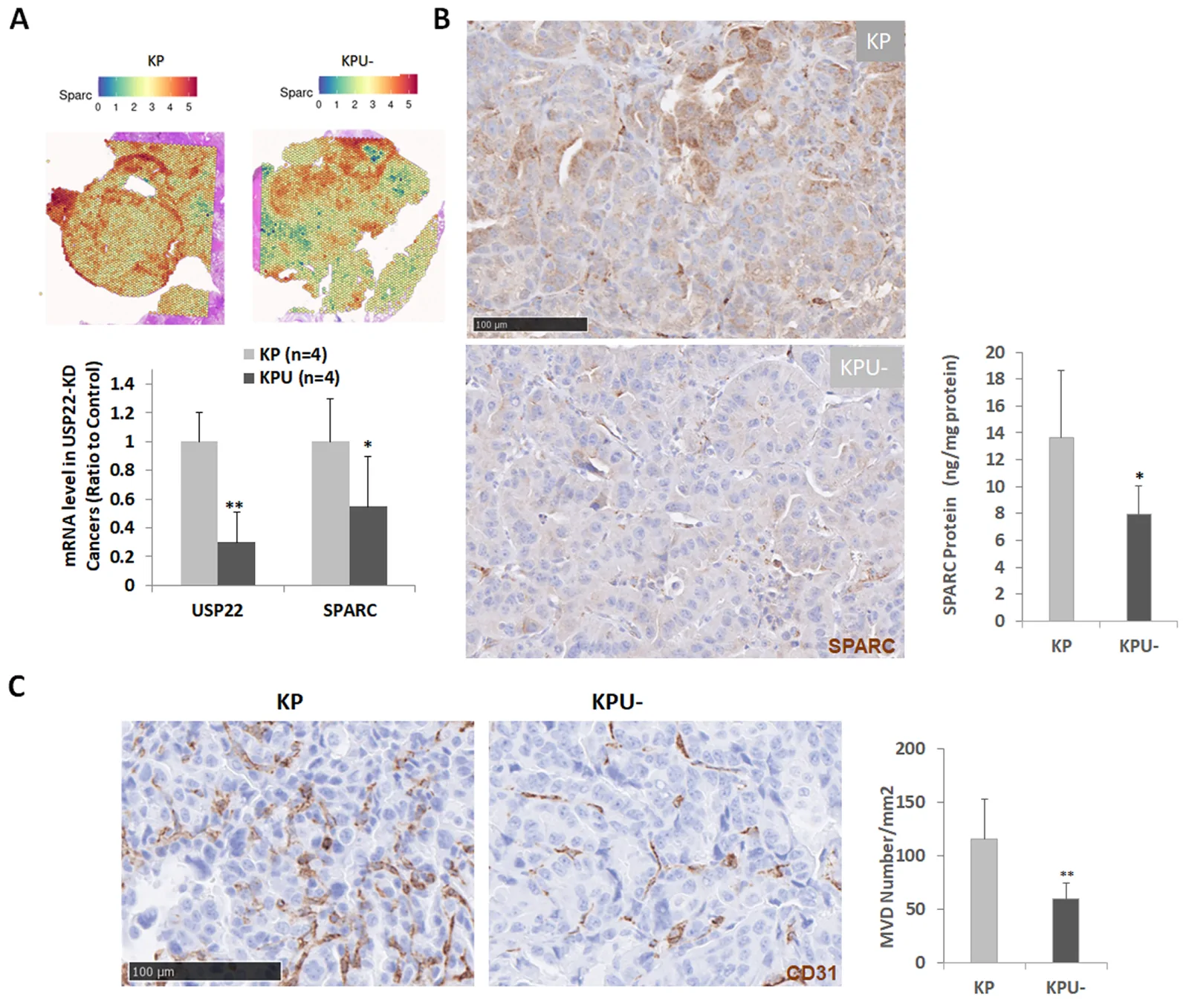
**Usp22-KO significantly decreased angiogenesis and SPARC in KP lung cancer.** (**A**) Decreased mRNA expression of SPARC. Left panel: spatial transcriptomics plots, right panel: quantitative RT-PCR of SPARC (n = 4), *P < 0.05, **P < 0.01, KP vs. KPU- lung cancer. (**B**) Decreased SPARC protein. Left panel: IHC staining of SPARC protein (brown, 200×). Right panel: quantitative ELISA analysis of SPARC protein in KP (n = 7) and KPU- (n = 7), *P < 0.05, KPU- vs. KP. (**C**) IHC of the endothelial marker CD31 (brown, 200× magnification). Semi-quantitative analysis of MVD in KP lung cancer tissues (n = 7) and KPU- lung cancer tissues (n = 7), **P < 0.01, KPU- vs. KP.

Both bulk and spatial RNA transcriptomic profiling analyses were employed to elucidate the mechanisms by which Usp22-KO reshapes the TME. Among the differentially expressed genes, SPARC was further examined due to its known roles in cancer. Both spatial transcriptomics and quantitative RT-PCR demonstrated a significant reduction in SPARC mRNA levels in KPU- lung cancers relative to parental KP cancers (**Figure 7A,** upper left). This decrease was corroborated at the protein level detected by IHC and ELISA, each showing markedly lower SPARC abundance in KPU- samples (**Figure 7B**, middle and right panels). We previously found that Usp22-KO diminishes angiogenesis in KRAS-driven lung cancer xenograft models 13, 28, and USP22 is a pro-angiogenic regulator in cancer 45, 46. In addition, given known pro-angiogenic functions of SPARC 47, 48, we next quantified MVD in KP and KPU- cancers using CD31 immunostaining. KPU- tumors exhibited a significant reduction in MVD, indicating impaired in vivo angiogenesis upon Usp22-KO (**Figure 7C**). Collectively, these data support a model in which Usp22-KO may regulate key ECM components, particularly SPARC, as well as angiogenesis in KP lung cancers, thereby potentially contributing to changes in the TME and immune cell infiltration.

### TGF-β1 and SPARC regulate macrophage migration and polarization

**Figure 8:**
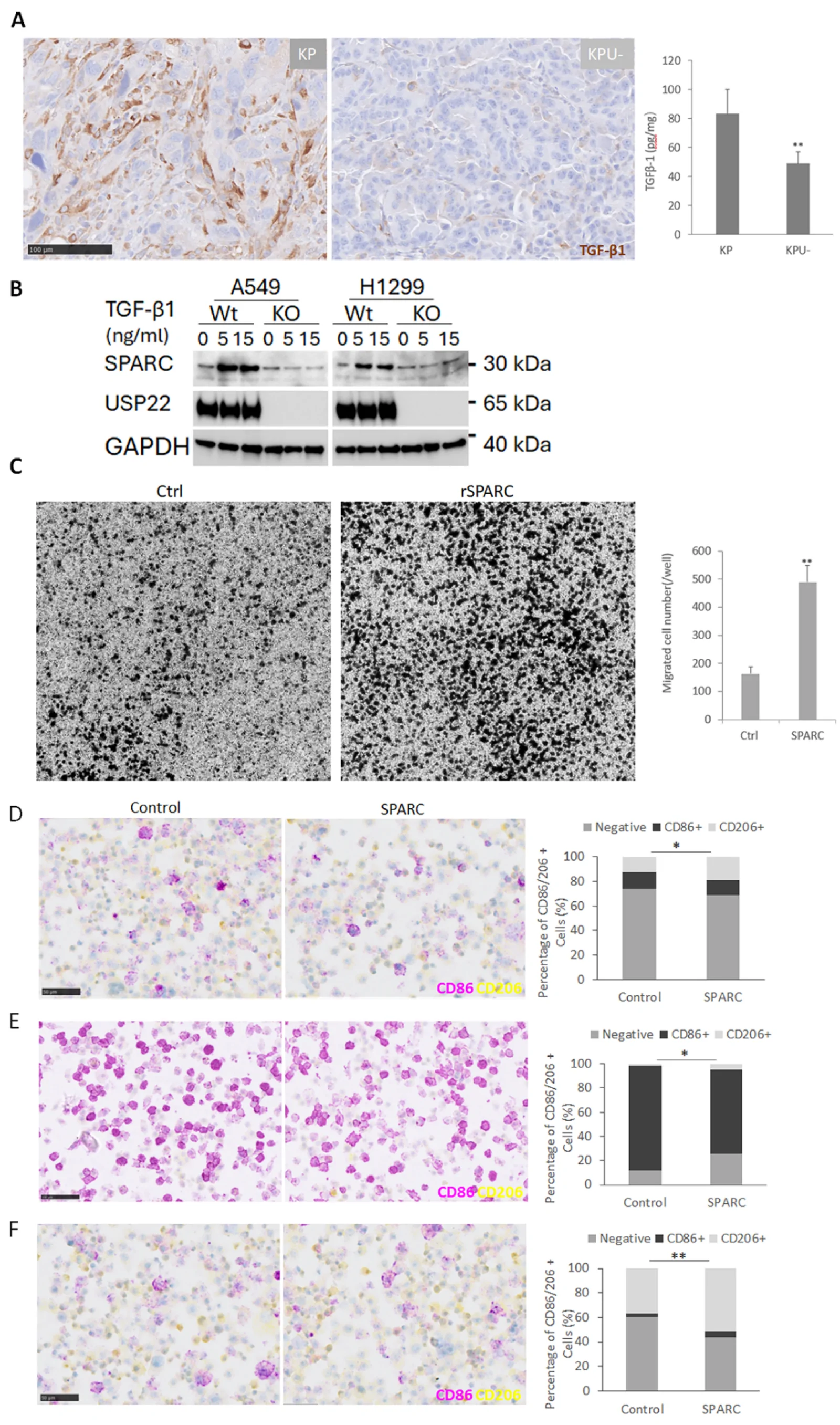
**Decreased TGF-β1 in KPU- lung cancer and its regulation on SPARC and macrophage migration and polarization.** (**A**) Decreased TGF-β1, left panel: IHC analysis of TGF-β1 (brown, scale bar: 50 µm, 200× magnification) in KP and KPU- lung cancers. Right panel: quantitative ELISA analysis of TGF-β1 in KP (n = 7) and KPU- (n = 7), **P < 0.01, KPU- vs. KP. (**B**) Western blot analysis of induced SPARC in USP22-KO and parental (Wt) Kras-mutant A549 and Hras-mutant H1299 lung cancer cells, cancer cells were treated for 48 h with 5 and 15 ng/mL of TGF-β1. (**C**) Increased migration of macrophage cell lines RAW264.7 treated with mouse recombinant SPARC (rSPARC, 500 µg/mL) for 24 h. Left panel: images of migrated RAW264.7 cells. Right panel: quantitative analysis of migrated cells in control (Ctrl), and rSPARC-treated RAW264.7 cells, **P < 0.01, rSPARC vs. Ctrl. (**D**) Quantification of CD86 (M1 marker, purple) and CD206 (M2 marker, yellow) co-IHC in RAW264.7 macrophage cells treated with control or rSPARC; right panel: marker-positive cell counts. (**E**) Quantification of CD86/CD206 in RAW264.7 cells treated with LPS/IFN-γ alone or combined with rSPARC. (**F**) Quantification of CD86/CD206 in cells treated with TGF-β alone or combined with rSPARC, *P < 0.05, **P < 0.01, rSPARC vs. Control.

The TME in Usp22-KO cancer is characterized by the markedly reduced infiltration of macrophages. To elucidate the underlying mechanisms, we focused on TGF-β1, SPARC due to their established roles in macrophage differentiation, migration, and immunoregulation. Spatial transcriptomic analysis revealed a significant downregulation of TGF-β1 expression in KPU- tumors. This reduction was further validated at the protein level by both IHC and ELISA (**Figure 8A**). Previous studies have demonstrated that TGF-β1 can induce SPARC expression in various cell types 49, 50. To investigate this regulation, we examined the potential of TGF-β1 to induce SPARC expression in lung cancer cells. TGF-β1 significantly upregulated SPARC expression in RAS-mutant lung cancer cells; notably, this induction was attenuated in Usp22-KO cells, suggesting that USP22 is required for the TGF-β1-mediated upregulation of SPARC (**Figure 8B**).

Given that SPARC has been implicated in regulating macrophage infiltration and polarization 51, we next assessed its functional role in macrophage behavior. To prove the concept, we first examined the effects of recombinant mouse SPARC (rSPARC) on the *in vitro* migration and induced polarization of mouse macrophage cell line RAW264.7. In vitro migration assays revealed that rSPARC significantly enhanced macrophage migration (**Figure 8C**). Additionally, SPARC promoted M2 polarization of RAW264.7 cells, as evidenced by the increased CD206-positive M2 subpopulation cells (**Figure 8D**). In contrast, SPARC significantly suppressed LPS plus IFN-γ-induced M1 polarization, as indicated by the reduced percentage of CD86-positive RAW264.7 cells compared with those treated with LPS plus IFN-γ alone (**Figure 8E**). Furthermore, SPARC enhanced TGF-β1-induced M2 polarization (**Figure 8F**).

Next, we compared the effects of conditioned media (CM) from mouse KP lung cancer cells (KP-CM) and KPU- cancer cells (KPU-CM) on macrophage migration. In time-course in vitro migration assays, KP-CM chemoattracted significantly more RAW264.7 macrophages than KPU-CM, with the greatest difference observed at 24 h (data not shown). Similarly, co-culture experiments showed that KPU- cancer cells recruited significantly fewer macrophages than KP cells (**Figure S7A**).

**Figure 9:**
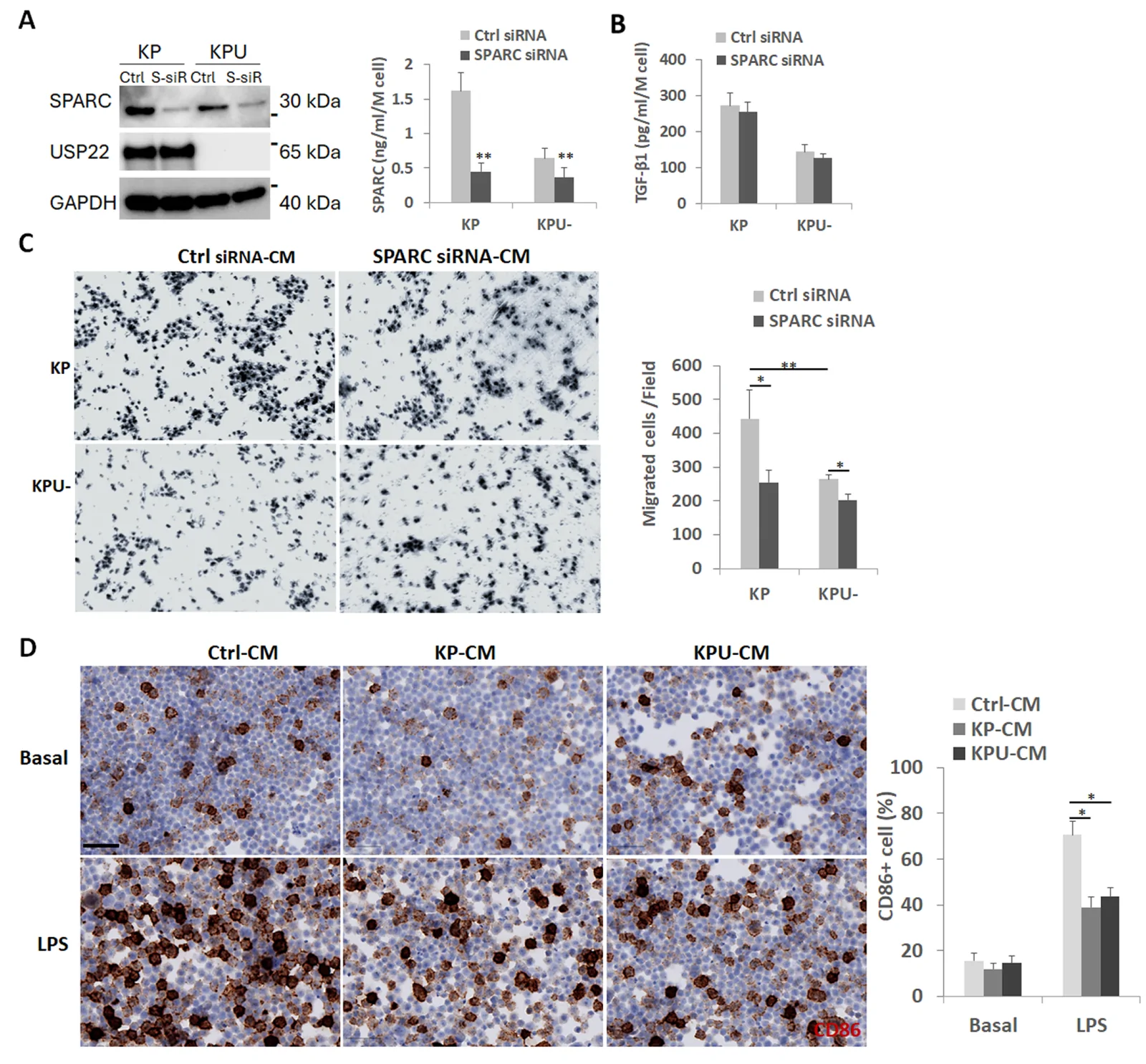

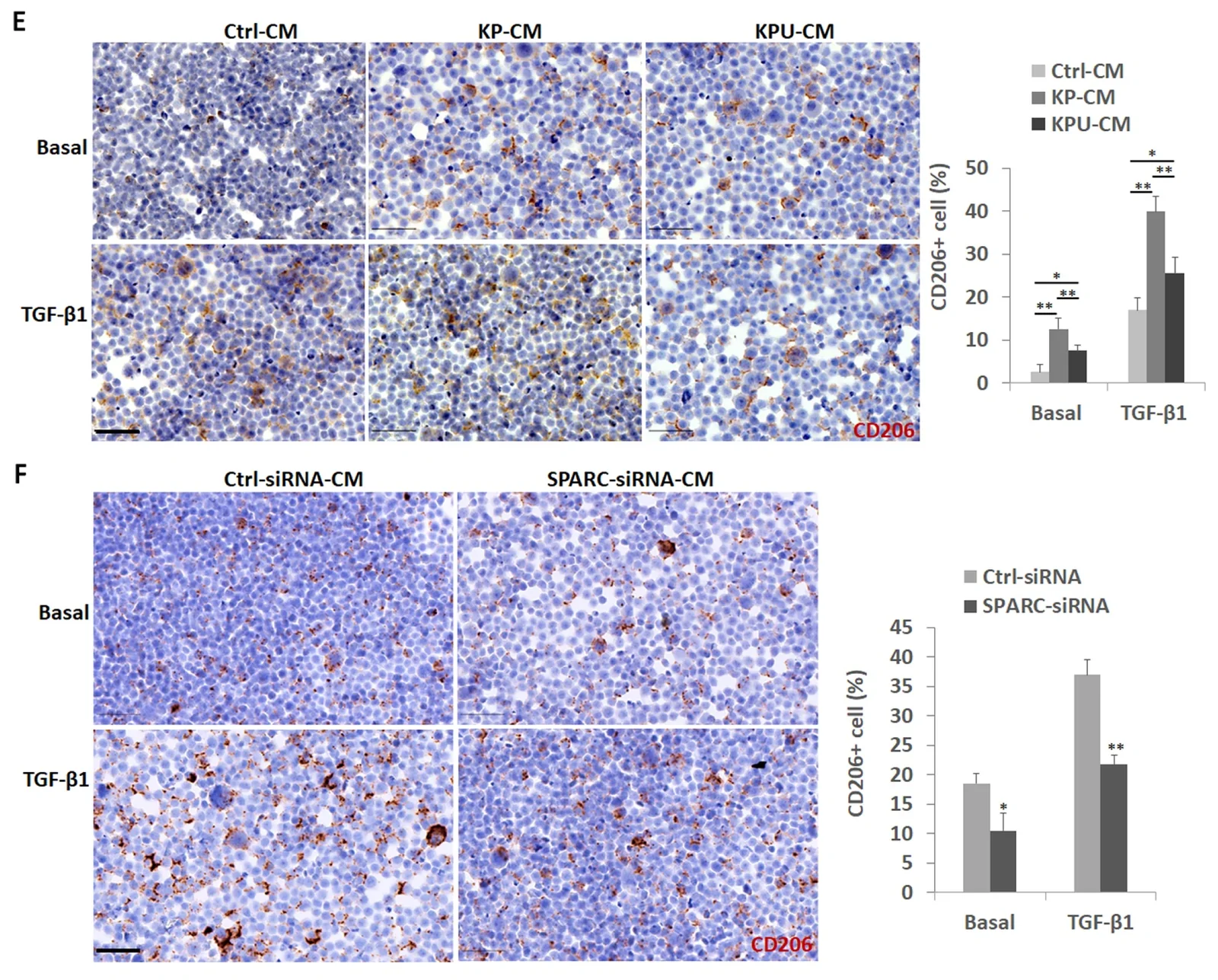
** Effects of KP-CM, KPU-CM, and SPARC knockdown on RAW264.7 macrophage migration and polarization.** (**A**) Western blot (left) and conditioned media (CM) ELISA (right; per million cells) showing SPARC expression in KP and KPU- cells transfected with control (Ctrl) or SPARC-targeting siRNA (S-siRNA); SPARC-siRNA-CM vs. Ctrl-siRNA-CM, **P < 0.01. (**B**) TGF-β1 levels in CMs from KP and KPU- cells transfected with control (Ctrl) or SPARC siRNA. (**C**) Chemotactic activity of CMs from KP and KPU- cells transfected with control or SPARC siRNA. Left: representative Transwell migration images of RAW264.7 cells exposed to CMs. Right: quantification of migrated cells, showing reduced chemotactic activity in KPU-CM and SPARC siRNA-CM compared with KP-CM and Ctrl siRNA-CM, respectively (*P < 0.05, **P < 0.01; SPARC siRNA vs Ctrl siRNA, KPU- vs. KP*).* (**D**) KP-CM and KPU-CM comparably suppressed LPS/IFN-γ-induced M1 polarization in RAW264.7 macrophages. Basal, unstimulated condition without LPS/IFN-γ. Left panel: representative CD86 IHC staining (M1 marker; scale bar: 50 μm; 200× magnification). Right panel: semi-quantitative analysis of the percentage of CD86-positive RAW264.7 cells, *P < 0.05, KP/KPU-CM vs. Ctrl-CM. (**E**) M2 polarization analysis of RAW264.7 cells cultured with CMs from KP or KPU- cells under basal conditions or in the presence of TGF-β1. Left panel: representative CD206 immunostaining (scale bar: 50 μm; 200× magnification). Right panel: quantification (percentage) of CD206+ cells, showing that KPU-CM induced weaker M2 polarization than KP-CM under both basal and TGF-β1 conditions, *P < 0.05, **P < 0.01; KP/KPU-CMs vs. Ctrl-CM, KPU-CM vs. KP-CM. (**F**) SPARC knockdown attenuates KP-CM-induced basal and TGF-β1-stimulated M2 polarization. Left panel: IHC staining of CD206 in RAW264.7 cells cultured with Ctrl-siRNA-CM or SPARC-siRNA-CM. Right panel: semi-quantitative analysis of the percentage of CD206-positive RAW264.7 cells; *P < 0.05, **P < 0.01; SPARC-siRNA-CM vs. Ctrl-siRNA-CM. Statistical analyses were performed using triplicate samples from two independent experiments.

We then investigated the contribution of SPARC to macrophage recruitment. As shown in **Figure 9A**, SPARC protein levels were significantly lower in KPU- cancer cells than in KP cells, and SPARC siRNA effectively reduced SPARC expression in both cancer cells and their corresponding CMs. TGF-β1 levels were lower in KPU-CM than in KP-CM but were not altered by SPARC knockdown in either cancer cell type (**Figure 9B**). Functionally, CM from SPARC-silenced KP cells attracted significantly fewer macrophages, whereas CM from SPARC-silenced KPU- cells further reduced macrophage migration (**Figure 9C**), while addition of rSPARC enhanced the macrophage-recruiting activity of KPU-CM (data not shown), consistent with the finding in Figure 8C.

Lastly, we examined the effects of KP-CM and KPU-CM on macrophage polarization. Compared with control CM (Ctrl-CM; culture medium used for CM collection), neither KP-CM nor KPU-CM markedly altered CD86-positive (M1) RAW264.7 cells under basal (unstimulated) conditions. However, both CMs comparably suppressed LPS/IFN-γ-induced M1 polarization (**Figure 9D**), indicating that inhibitory factors present in CM predominate under these conditions and that SPARC is unlikely to be the major mediator of this effect; this finding is consistent with the presence of rare but comparable M1 macrophage infiltration in both KP and KPU- lung cancers. Reducing CM volume in culture medium attenuated the suppression of LPS/IFN-γ-induced M1 polarization (**Figure S7B**), supporting a dose-dependent effect.

Although both KP-CM and KPU-CM increased basal and TGF-β1-induced CD206-positive M2 polarization in RAW264.7 macrophages relative to Ctrl-CM, KPU-CM exhibited a significantly lower enhancement of M2 polarization than KP-CM (**Figure 9E**). Addition of lower CM volumes also attenuated M2 polarization (**Figure S7C**), further supporting a dose-dependent response.

To further assess the role of SPARC, RAW264.7 cells were treated with TGF-β1 in the presence of CM from control or SPARC-knockdown KP and KPU- cells. SPARC knockdown significantly reduced the ability of CM to promote both basal and TGF-β1-induced CD206-positive M2 polarization (**Figure 9F**). Consistently, supplementation with rSPARC enhanced the M2-promoting activity of CM from KPU- cells, whereas SPARC knockdown in KPU- cells further slightly reduced the effect of its CM on M2 polarization (data not shown), which is consistent with the finding in Figure 8F.

In addition to SPARC, we also assessed MCP-1 (CCL2), a chemokine known to recruit monocytes, macrophages, and other immune cells 52. Although a slight reduction in MCP-1 protein levels was observed in KPU- tumors compared to KP tumors, the difference did not reach statistical significance (**Figure S8**).

In summary, our findings suggest that USP22 contributes to the regulation of the TME through modulation of the two interregulated proteins TGF-β1 and SPARC, thereby modulating immune cell infiltration, with a specific effect on migration and M2 polarization of macrophages, and potentially promoting tumor immune evasion and progression. These results underscore the potential of targeting USP22 as a strategy to remodel the TME and enhance antitumor immunity.

### Positive correlation of USP22 with SPARC in human lung cancer tissues

**Figure 10:**
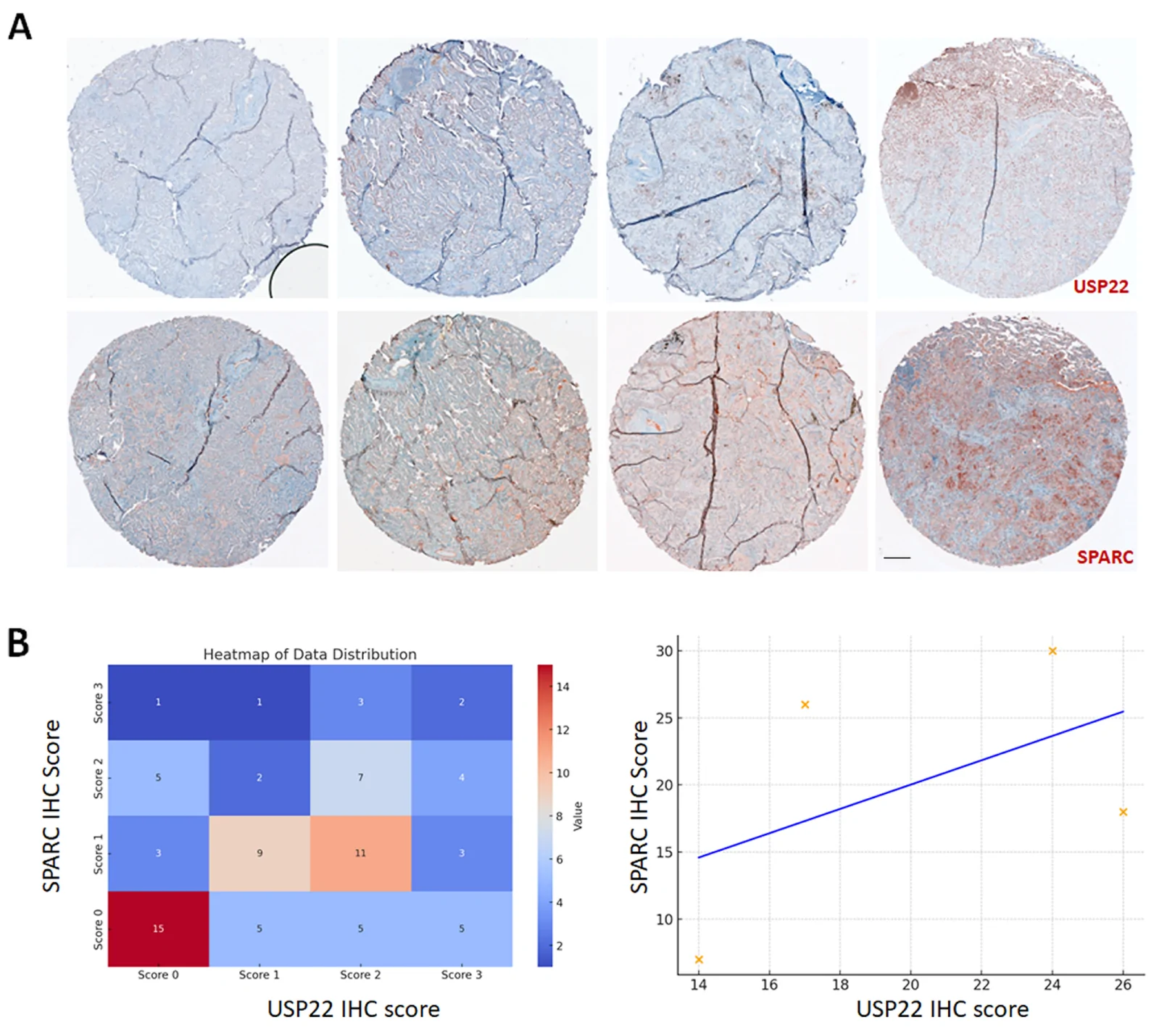
**Correlation between USP22 and SPARC IHC staining in human lung cancers**. (**A**) IHC images (scale bar: 500 μm, 30× magnification) of USP22 (upper panel) and SPARC (lower panel) in four representative tissues. (**B**) Correlation analysis of the co-expression of USP22 and SPARC. Left panel: Heatmap showing the distribution of cases according to USP22 IHC scores (0 - 3, x-axis) and SPARC IHC scores (0 - 3, y-axis). Numbers within cells indicate case frequencies, and color intensity reflects sample density. Right panel: Scatter plot with Pearson correlation analysis demonstrating a moderately positive association between USP22 and SPARC IHC scores (R = 0.51).

Our previous research demonstrated that USP22 is frequently overexpressed in human lung cancer 13-15. Building on these findings, the current study investigated whether SPARC could serve as a key downstream target of USP22 in lung cancer progression. Given SPARC’s known involvement in extracellular matrix regulation, TGF-β signaling activation, and immune cell recruitment, we specifically considered its potential role in USP22-mediated TGF-β activation and macrophage infiltration. To explore this relationship, we analyzed the expression patterns of both USP22 and SPARC in the same lung cancer tissue microarray (TMA). Our results revealed that SPARC was overexpressed in more than 60% of lung cancer tissue samples, as illustrated in **Figure 10A**. Representative tissue sections showed concurrent expression of USP22 and SPARC within the same tumors. To further evaluate their association, we performed Pearson correlation analysis using the expression data from these samples. The analysis demonstrated a moderate positive correlation between USP22 and SPARC (R = 0.51), as shown in **Figure 10B**. The left panel presents a heatmap of the expression distribution, while the right panel displays the corresponding correlation curve. These findings suggest that SPARC expression may be regulated, at least in part, by USP22 and could play an important role in USP22-driven immunosuppressive mechanisms in lung cancer.

Analysis of IHC scores revealed a heterogeneous distribution of USP22 and SPARC expression levels. The highest frequency of cases was observed in the USP22 score 0/SPARC score 0 category (n = 15), followed by USP22 score 2/SPARC score 1 (n = 11) and USP22 score 1/SPARC score 1 (n = 9). Overall, USP22 score 2 represented the most prevalent USP22 expression category, whereas SPARC score 1 was the most frequently observed SPARC category. Visualization of the score distribution demonstrated a tendency for higher SPARC expression to occur in samples with higher USP22 expression. Consistent with this observation, linear regression analysis showed a positive slope between USP22 and SPARC IHC scores, indicating a positive association between the two markers. However, the dispersion of data points suggests that the relationship is moderate and may require statistical correlation testing (e.g., Spearman's rank correlation) to determine significance.

## Discussion

USP22 is overexpressed in multiple tumor types and is linked to cancer stemness, chemotherapy resistance, and poor prognosis in cancers such as hepatocellular, breast, and colorectal cancers 7, 45, 53-55. Recent studies further show that USP22 shapes the TME by stabilizing PD-L1 on cancer cells 16, 17 and enhancing Foxp3 activity in Tregs 16, 17, thereby suppressing antitumor immunity. In this study, we demonstrate that Usp22-KO significantly inhibits tumorigenesis and alters immune cell infiltration in KP mouse lung cancer, characterized by increased cytotoxic CD8+ T cells and reduced Tregs and M2 macrophages. These findings suggest that targeting USP22 may reprogram the TME through cancer–immune cell crosstalk, potentially sensitizing tumors to immunotherapy.

Our findings reveal that Usp22-KO effectively suppresses *in vivo* cancer growth in KP lung cancer, aligning with its established role in regulating cell-cycle activity. Mechanistically, USP22 stabilizes or regulates key oncogenic proteins, including SIRT1, c-Myc, and CCND1, while inhibiting several p53-mediated tumor-suppressive functions 18. Its knockdown has been shown to reduce cancer cell proliferation and tumor growth across various models 8-10. In liver cancer, USP22 depletion suppresses tumor progression and enhances the efficacy of cisplatin-based chemotherapy in mice 8-12. Our recent studies further demonstrate that USP22 knockdown disrupts cancer stem cell maintenance, angiogenesis, tumor growth, and metastasis in KRAS-mutant lung cancer, while also sensitizing tumors to cisplatin and radiation therapy, independent of p53 status 16, 17. In addition to its direct effects on cancer cells, Usp22-KO may also influence anticancer immune responses, further contributing to the observed suppression of tumor growth.

The KRAS oncogene plays a pivotal role in modulating the immune response by reshaping the TME in lung cancer. KRAS-mutant tumors often exhibit immunosuppressive TME, marked by reduced infiltration of cytotoxic CD8+ T cells and an increase in Tregs 13-15, which dampen antitumor immunity. A high density of Foxp3+ tumor-infiltrating Tregs correlates with increased recurrence and poor overall survival in KRAS-mutant NSCLC 25. Moreover, KRAS activation upregulates immune checkpoint molecules like PD-L1, contributing to immune evasion. Oncogenic KRAS stabilizes PD-L1 mRNA 43, promoting cancer-TME crosstalk, inflammation, and immunosuppression, thereby facilitating tumor progression, invasion, and metastasis 44. Elevated PD-L1 further induces apoptosis of CD3+ T cells, exacerbating immunosuppression and therapy resistance 45, 46. In this study, we show that USP22 knockout significantly reduces PD-L1 expression on cancer cells and alters immune cell infiltration, increasing CD8+ T cells while decreasing Tregs, MDSCs, and macrophages. In KRAS-mutant NSCLC, USP22 depletion disrupts these immunosuppressive mechanisms, highlighting its critical role in tumor progression. This finding is generally consistent with a previous study that reported that deletion of USP22 in pancreatic tumor cells reduced the infiltration of MDSC and promoted the infiltration of CD4+ and CD8+ T cells and NK cells, leading to an improved response to combination immunotherapy 40, while the abundance of NK cells, which is markedly low in KP cancer, remained unchanged, indicating a tumor-type-specific response. Interestingly, a recent study shows that USP22 promotes tumor immune evasion and resistance to ICB by suppressing MHC-I expression. Notably, higher USP22 levels are strongly associated with reduced CD8+ T-cell infiltration and broader impairment of antitumor immune cell activity. In patients with lung cancer, USP22 upregulation correlates with poor response to ICB 56, underscoring its clinical relevance as a driver of immune exclusion and therapeutic resistance. Notably, targeting USP22 has been shown to enhance the efficacy of PD-L1-targeted immunotherapy, suggesting its potential to reverse TME-driven immune evasion 16, 17. In Usp22-KO KRAS-mutant lung tumors, the TME is characterized by a moderate increase in cytotoxic T cells and a marked reduction in M2 macrophages. This is particularly significant, as cytotoxic T cells are essential for effective antitumor immunity, and their absence is a major barrier in KRAS-driven lung cancer. Tumor-infiltrating T cells are strong predictors of immunotherapy response, with T-cell inflamed tumors showing better outcomes following ICI 57. Preclinical data further link activated CD8+ T cells to improved therapy sensitivity 58. Emerging evidence underscores the role of tumor-intrinsic factors, such as signaling pathways, secreted molecules, and epigenetic regulators, in shaping the immune TME 59. Among these, USP22 stands out as a key regulator of TME composition and dynamics, offering new insights into how tumor-intrinsic epigenetic mechanisms contribute to immune modulation.

Immune checkpoint inhibitors targeting PD-1/PD-L1 have become a cornerstone in treating advanced KRAS-mutant NSCLC 12. However, most patients do not respond to these therapies, and responses are often short-lived 4, 21. This highlights the urgent need for new strategies for KRAS-mutant lung cancer. In this study, we demonstrate that Usp22-KO significantly sensitizes KP lung cancer to ICI. Several mechanisms may underlie this effect. First, USP22 directly deubiquitinates and stabilizes PD-L1 on cancer cells, enhancing immune evasion 16, 17. Second, Usp22-KO increases cytotoxic T cell infiltration while reducing immunosuppressive Tregs and M2 macrophages 16, 17, collectively promoting a more favorable immune TME. By disrupting PD-L1 stabilization and immunosuppressive cell recruitment, USP22 inhibition enhances T-cell–mediated antitumor responses and improves sensitivity to ICI. These findings are consistent with reports in liver and pancreatic cancers, where USP22 similarly regulates immune infiltration and immunotherapy response 18, 60.

The interaction between tumor cells and the surrounding stroma is critical for cancer growth, differentiation, progression, and metastasis, ultimately influencing tumor aggressiveness 61. In this context, Usp22-KO appears to modulate the TME through multiple mechanisms that promote antitumor immunity. Consistent with our prior findings, Usp22-KO alters several signaling pathways in cancer cells. Importantly, it also affects stromal components of the TME, including fibroblasts and immune cells, thereby contributing to tumor suppression. In this study, we observed that Usp22-KO significantly reduced levels of TGF-β1, a key cytokine known to facilitate epithelial-to-mesenchymal transition (EMT), invasion, metastasis, and immune evasion 39. Consistently, a previous study showed that USP22 upregulates TGF-β1, and that there is a strong positive correlation between USP22 and TGF-β1 in NSCLCs 62. TGF-β1 is a well-characterized immunosuppressive cytokine that contributes to multiple tumor-promoting processes. This finding is consistent with a previous study, which showed that USP22 promoted EMT and TGF-β1 was elevated in lung adenocarcinomas with high USP22 protein expression, and lung adenocarcinomas expressing both USP22 and TGF-β1 were associated with a poorer prognosis 62. Similarly, another study revealed that Usp22-KO in mice results in embryonic lethality due to failure of vasculature formation through defective kinase signaling including TGF-β and several receptor tyrosine kinase pathways 45. In addition, Usp22-KO decreased expression of SPARC, an ECM protein implicated in ECM remodeling, angiogenesis, and tumor cell adhesion. A previous study reported that USP22 transcriptionally downregulated SPARC in acute colitis and inflammation-associated colorectal cancer, whereas our findings demonstrate the opposite effect of USP22 on SPARC transcription 63; this discrepancy suggests that USP22-mediated regulation of SPARC may be tissue- or context-dependent. SPARC is frequently overexpressed in lung cancer stroma and is associated with poor prognosis due to its role in promoting invasion and metastasis 51. Notably, TGF-β1 can induce SPARC expression in various cell types 49, 50. Conversely, SPARC has also been reported to modulate the expression and activity of the TGF-β1 signaling pathway in cancer 64, 65. Herein, we demonstrated that TGF-ß1 also upregulated SPARC in human RAS-mutant lung cancer cells, and interestingly, this induction was abolished in Usp22-KO cells. This suggests that USP22 is required for the TGF-β1-mediated upregulation of SPARC. SPARC has been implicated in regulating macrophage infiltration and polarization 51. We revealed that SPARC significantly enhanced in vitro migration of macrophages. Tumor-associated macrophages (TAMs) within the TME promote tumor progression by interacting with various cells to regulate the M1/M2 balance and foster an immunosuppressive milieu, leading to immune evasion. As a major component of the TME, TAMs significantly impair the efficacy of PD-1/PD-L1 inhibitors and contribute to immunotherapy resistance 66. These findings indicate that the TGF-β1-SPARC axis plays a critical role in promoting macrophage infiltration into the TME. Disruption of this axis in Usp22-KO tumors may impair the positive feedback loop between TGF-β1 and SPARC, thereby attenuating immune suppression and altering immune cell composition within the TME of KP tumors.

Notably, SPARC also regulates macrophage infiltration and polarization 51, which may explain the observed reduction in M2 macrophages in Usp22-KO tumors compared to parental KP lung cancers. Furthermore, USP22 is known to stabilize c-Myc by deubiquitinating 44. In addition, TGF-β1 67 and VEGF, which are upregulated by USP22 in cancer 46, may contribute to the induction of PD-L1 expression in M2 TAMs. In this study, Usp22-KO in KPU- resulted in a significant reduction in PD-L1+ M2 macrophages. PD-L1+ TAMs exert potent immunosuppressive effects by engaging PD-1 on CD8+ T cells and activating SHP2-mediated signaling, which inhibits key downstream pathways including Syk, PI3K, AKT, mTOR, and ERK2. This leads to reduced T cell proliferation, survival, cytokine secretion (e.g., IFN-γ and TNF-α), and ultimately T cell apoptosis—diminishing antitumor immunity. Compared to their PD-L1- counterparts, PD-L1+ M2 macrophages are more effective at suppressing T cell function and promoting immune evasion 41, 68, and their abundance is associated with poor prognosis in cancer patients. Furthermore, transplantation of autocrine VEGF-stimulated PD-L1+ M2 macrophages into allogeneic mice significantly suppressed peripheral CD4+ and CD8+ T cells and increased CD4+CD25+ Tregs in the bone marrow, reinforcing their systemic immunosuppressive role 67. In NSCLC, TAM enrichment in the TME correlates with resistance to immune checkpoint blockade 69, highlighting the therapeutic potential of targeting PD-L1+ TAMs to enhance the efficacy of immunotherapy.

Co-occurring mutations in *KRAS* and c*-Myc* synergistically drive lung tumorigenesis by promoting an inflammatory, angiogenic, and immunosuppressive TME, as shown in transgenic mouse models 70. Angiogenesis inhibition is well established to reshape the TME and, consequently, to influence immune cell infiltration and the efficacy of ICB 71. In this context, we found that Usp22-KO suppresses angiogenesis, consistent with the recognized role of USP22 as a pro-angiogenic regulator via enhancement of the HIF-1α-VEGF axis 45, 46. Therefore, the loss of USP22 likely alters the TME by reducing angiogenesis, which in turn may modulate immune infiltration and impact responses to ICB. Therefore, Usp22-KO may reduce c-Myc, VEGF, and SPARC levels, potentially contributing to diminished angiogenesis and a reshaped TME, as well as altered immune infiltration including MDSCs and macrophage recruitment, thereby creating a less immunosuppressive environment. Overall, these findings suggest that USP22 regulates the TME through multiple tumor-intrinsic and stromal pathways. Its depletion not only inhibits oncogenic signaling but also reprograms the immune landscape, thereby enhancing tumor sensitivity to ICI. This highlights USP22 as a promising therapeutic target for modulating the TME and improving immunotherapy outcomes. In hepatocellular carcinoma, USP22 was positively associated with CD68+ TAM and poor prognosis of patients 72.

Despite the transformative impact of ICIs in NSCLC, most patients, particularly those with KRAS mutations co-occurring with liver kinase B1/serine and threonine kinase 11 (LKB1/STK11) mutations either do not respond or exhibit only transient responses 4, 21. Combining ICIs with chemotherapy or other immunotherapies has improved some outcomes. For instance, the recently approved KRAS G12C inhibitor AMG 510 showed enhanced antitumor efficacy and favorable TME modulation when combined with ICIs in preclinical models, and a clinical trial is currently evaluating this combination 73. Given the lack of effective treatment options for many patients with KRAS-mutant tumors, especially KL-type lung cancer, targeting USP22 represents a novel and promising strategy to overcome immune resistance and improve the efficacy of immunotherapy in NSCLC. However, a limitation of this study is that it solely examined the impact of USP22 in KP lung cancer; therefore, the potential role of USP22 in KL lung cancer will be further investigated in future studies.

## Conclusions

**Figure 11:**
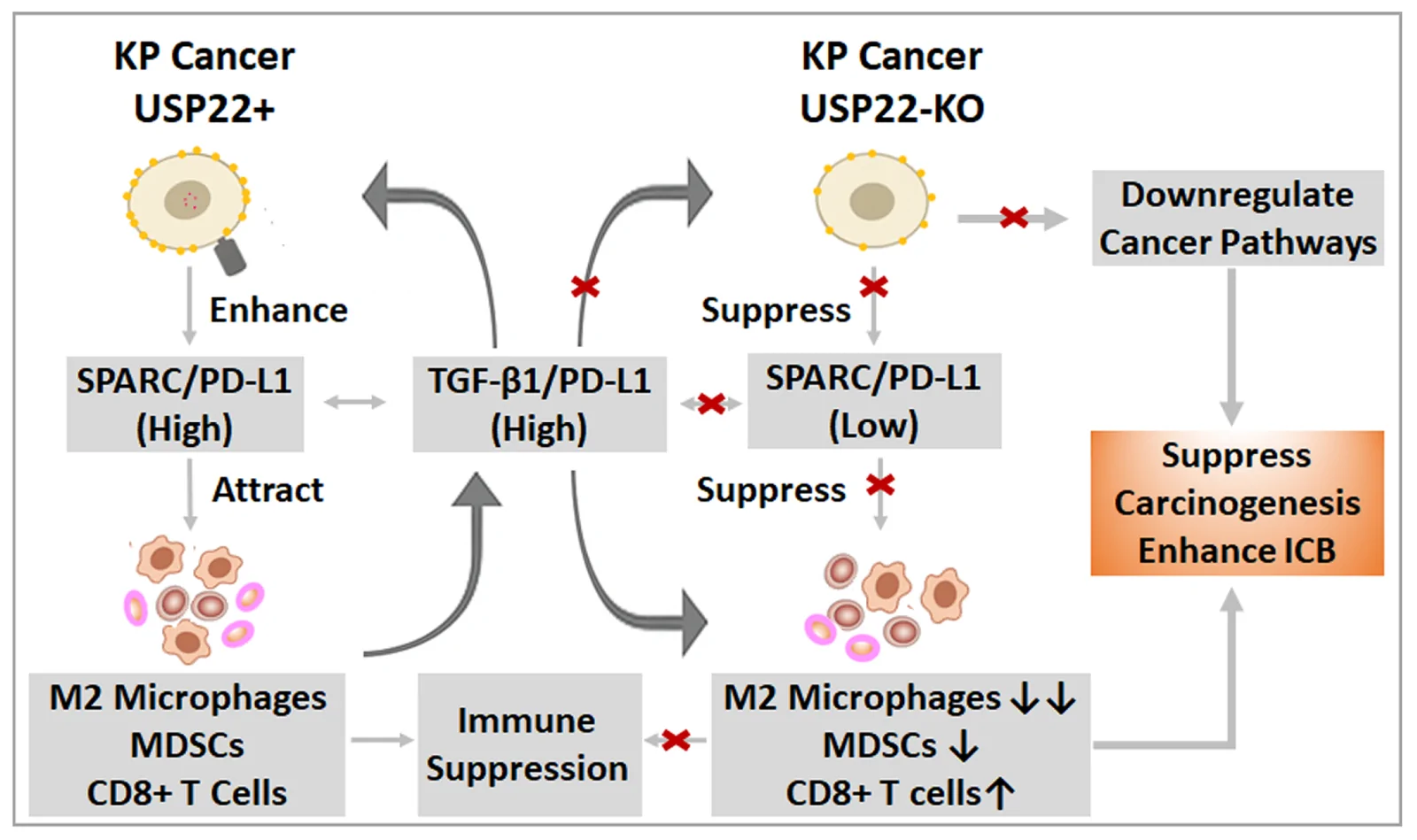
** Summary schematic model of the potential role of USP22 in shaping the TME of KP lung cancer and its impact on ICI therapy.** In USP22-highly expressed KP lung cancers, elevated levels of SPARC, PD-L1, c-Myc, and TGF-β1 contribute to an immunosuppressive TME. This milieu promotes the recruitment and polarization of immunosuppressive M2 macrophages and MDSCs, which collectively inhibit CD8+ T cell activation and function, thereby facilitating immune evasion and cancer progression. Usp22-KO leads to downregulation of SPARC, PD-L1, and c-Myc, along with reduced TGF-β1 production. These changes are associated with decreased infiltration of M2 macrophages and MDSCs and a corresponding enhancement of CD8+ T cell-mediated anti-tumor immunity. Together, this reprogramming of the TME suppresses carcinogenesis and improves the efficacy of ICI therapy.

In summary, this study demonstrates that Usp22-KO not only suppresses tumorigenesis in KP lung cancer but also profoundly remodels the TME. Usp22-KO regulates key signaling pathways and enhances tumor-stroma crosstalk, leading to reduced myeloid cell infiltration, increased cytotoxic T cell presence, and decreased Tregs and macrophages. These changes improve the response to immunotherapy (**Figure 11**). Our findings identify USP22 as a critical regulator of immune infiltration and immunotherapy sensitivity, highlighting its potential as a therapeutic target to overcome resistance and reprogram the TME in KRAS-mutant lung cancer.

## Supplementary Material

Supplementary figures.

## Data Availability

The data set supporting the conclusions of this article is included within the article and its additional file. RNA-Seq data is publicly available and accessible through NCBI GEO under accession numbers GSE306017 (RNA-seq) and GSE306019 (Visium).

## Abbreviations

Ad-Cre: adenovirus expressing Cre recombinase
CCND1: Cyclin D1
CSC: Cancer stem cell
DUBs: Deubiquitinases
ECM: Extracellular matrix
ELISA: Enzyme-linked immunosorbent assay
FFPE: Formalin-fixed, paraffin-embedded
GSEA: Gene-set enrichment analysis
H&E: Haematoxylin and Eosin
H2Bub1: Mono-ubiquitin moiety from lysine 120 of H2B
HIF-1α: hypoxia-inducible factor 1-alpha
IFN-γ: interferon-gamma
IHC: Immunohistochemistry
ICI: Immune checkpoint inhibitor
KP: KRAS^G12D^*-*LSL-p53^Flox/Flox^
*KPU-*: *KRAS*^G12D/+^-p53^Flox/Flox^- USP22^Flox/Flox^
LKB1/STK11: Liver kinase B1/serine and threonine kinase 11
LPS: lipopolysaccharide
MCP-1/CCL2: monocyte chemoattractant protein-1/ C-C motif chemokine ligand 2
MDSC: myeloid-derived suppression cell
mIF: Multiplex immunofluorescence
micro-CT: micro-computed tomography
MVD: Micro vessel density
NSCLC: Non-small cell lung cancer
PD-1: Programmed death-1
PD-L1: Programmed death-ligand 1
Sirt1: NAD-dependent protein deacetylase sirtuin-1
SPARC: Secreted protein acidic and rich in cysteine
TGF-β1: Transforming growth factor beta 1
TAM: Tumor-associated macrophage
TME: Tumor microenvironment
Tregs: T regulatory cells
UPS22: Ubiquitin-specific protease 22
Usp22-KO: USP22-knockout
VEGF: Vascular endothelial growth factor
