# Supplementary material for: Targeting USP22 reprograms the tumor microenvironment and sensitizes KRAS/p53-driven lung cancer to anti-PD-1 immunotherapy
Source: Theranostics. 2026 Aug 12;16(15):8708–32. doi: 10.7150/thno.125898 (PMC13549226; doi:10.7150/thno.125898)
Supplement: Supplementary file 1 [file thnov16p8708s1.pdf]

Targeting USP22 reprograms the tumor microenvironment and sensitizes KRAS/p53-driven lung tumor to anti-PD1 immunotherapy

Keqiang Zhang[1*], Ching Ouyang[2], Jonathan Castillo[3], Jinhui Wang[2], Walter Tsark[4], Yuanyuan Gao[1], Wendong Li[1], Minxiao Yang[3], Aimin Li[5], Britney Oeung[6], Colt Egelston[6], Jun Wu[7], Mahima Raul[1], Leonidas Arvanitis[8], Crystal Marconett[3], Dan J Raz[1*]

[1]Division of Thoracic Surgery, City of Hope National Medical Center, Duarte, California, USA
[2]Integrative Genomics Core, City of Hope Comprehensive Tumor Center, Duarte, CA, USA
[3]Department of Integrative Translational Sciences, City of Hope National Medical Center, Duarte, CA, USA
[4]Transgenic/Knockout Program, City of Hope National Medical Center, Duarte, CA, USA
[5]Pathology Core of Shared Resources, City of Hope National Medical Center, Duarte, CA, USA
[6]Immuno-Oncology Facility, Department of Immune-Oncology, City of Hope National Medical Center
[7]Division of Comparative Medicine, City of Hope National Medical Center, Duarte, CA, USA
[8]Department of Pathology, City of Hope National Medical Center, Duarte, CA, USA

Grant support
Dr. Raz was supported by the Department of Defense (USA) Lung Tumor Research Program grant Raz (W81XWH-22-1-0306), Dr. Marconett is supported by NCI grant (R01 CA262258). All cores are supported by the City of Hope Core grant from NCI P30CA33572.

Corresponding Authors
Dan J. Raz, M.D, Professor
City of Hope National Medical Center, 1500E Duarte Road, Duarte, CA 91010.
Phone: 626-278-7721; E-mail: draz@coh.org
Keqiang Zhang, Ph.D, Associate Research Professor
Phone: 626-218-4178; E-mail: kzhang@coh.org

Supplementary Figure 1

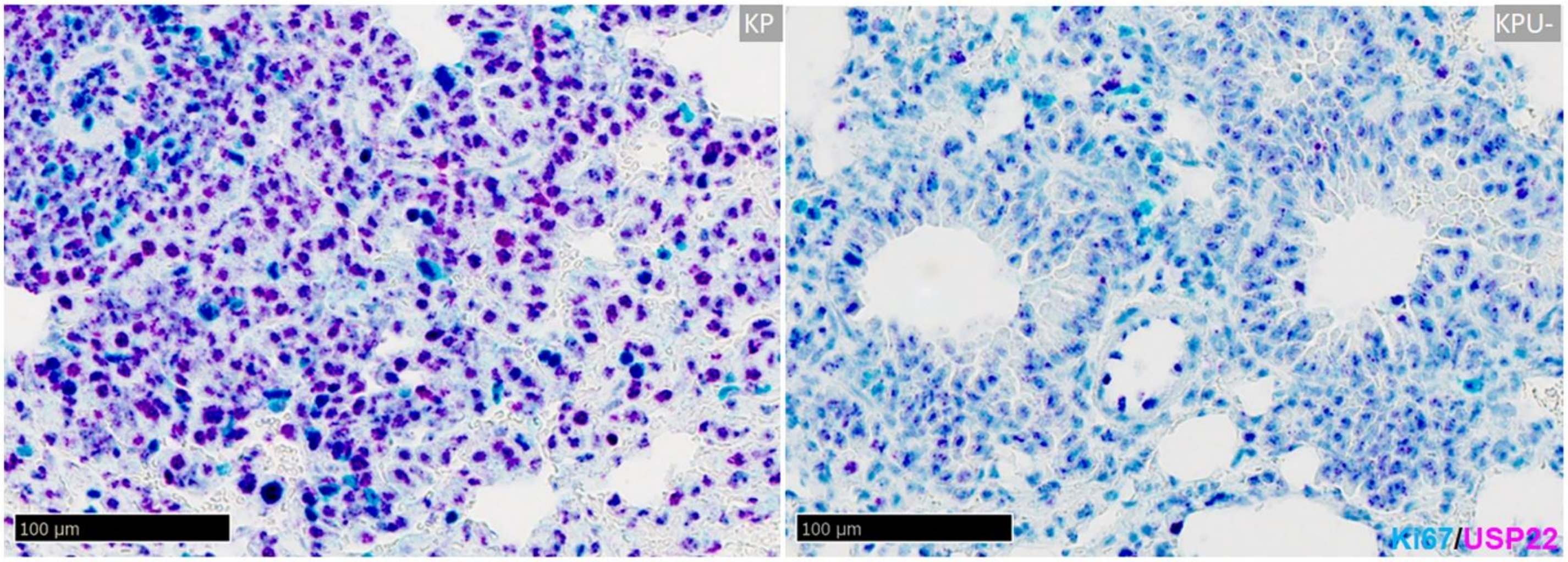

Figure S1. IHC staining images (scale bar: 100 μm, 200××x Magnification) of USP22 (purple) and Ki67 (cyan) in KP and KPU- lung tumors at 8 weeks after administration of Ad-Cre.

Supplementary Figure 2

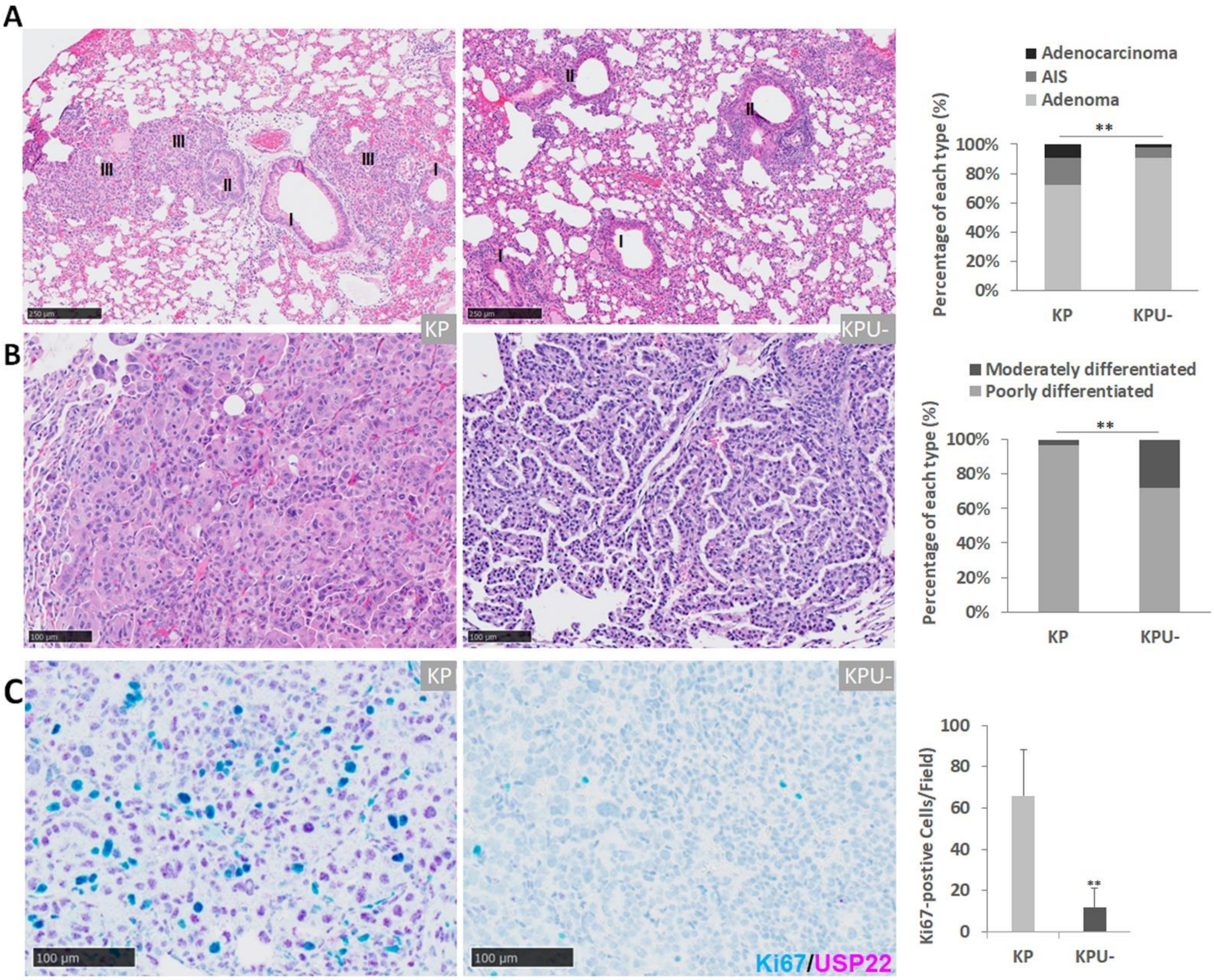

Figure S2. Usp22-KO suppressed carcinogenesis and differentiation of KRAS-G12D/p53-null mouse lung tumor. (**A**) H&E staining images (scale bar: 100 µm, 100× magnification) of KP and KPU- tumor tissues at 8 weeks of past-administration of Ad-Cre (left panel), areas labeled I as representative lesions of adenoma, II as lesions of adenocarcinoma in situ (AIS), and III as lesions of adenocarcinoma. Right panel: quantitative analysis of adenoma, AIS, and adenocarcinoma in KP and KPU- lung tumors (n = 7, $^{**}P < 0.01$, KPU- vs. KP). (**B**) H&E images (scale bar: 100 µm, 100× magnification): representative poorly differentiated adenocarcinomas in KP and moderately differentiated adenocarcinomas in KPU- tissue; right panel: quantitative analysis of each type of adenocarcinoma in KP and KPU- samples (n = 7, $^{**}P < 0.01$, KPU- vs. KP). (**C**) IHC staining images (scale bar: 100 µm, 200× magnification) of USP22 (purple) and Ki67 (cyan) in KP and KPU- lung tissues at 16 weeks after administration of Ad-Cre (left panel) and semiquantitative analysis of Ki67-positive tumor cells (n = 7, $^{**}P < 0.01$, KPU- vs. KP).

Supplementary Figure 3

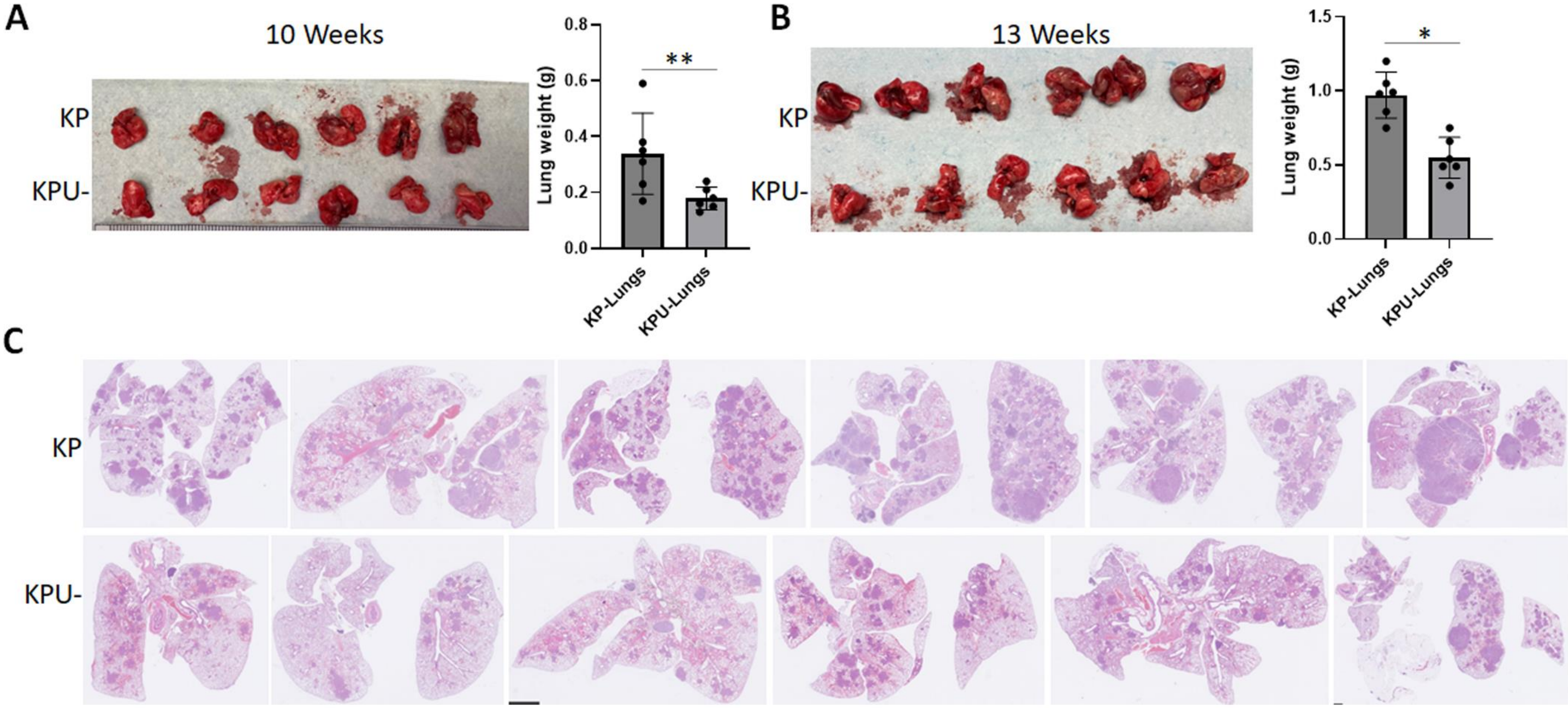

Figure S3. Gross lungs and H&E staining images of KP and KPU- lung tumors. Images of representative gross lungs and the wet weights of whole lungs at (**A**)10 and (**B**)13 weeks after tumor initiation, n = 6, *$P < 0.05$, **$P < 0.01$, KPU- versus KP. (**C**) H&E staining images of tumor areas in KP and KPU- mouse lungs (12 weeks post-initiation), which were stained deep blue.

Supplementary Figure 4

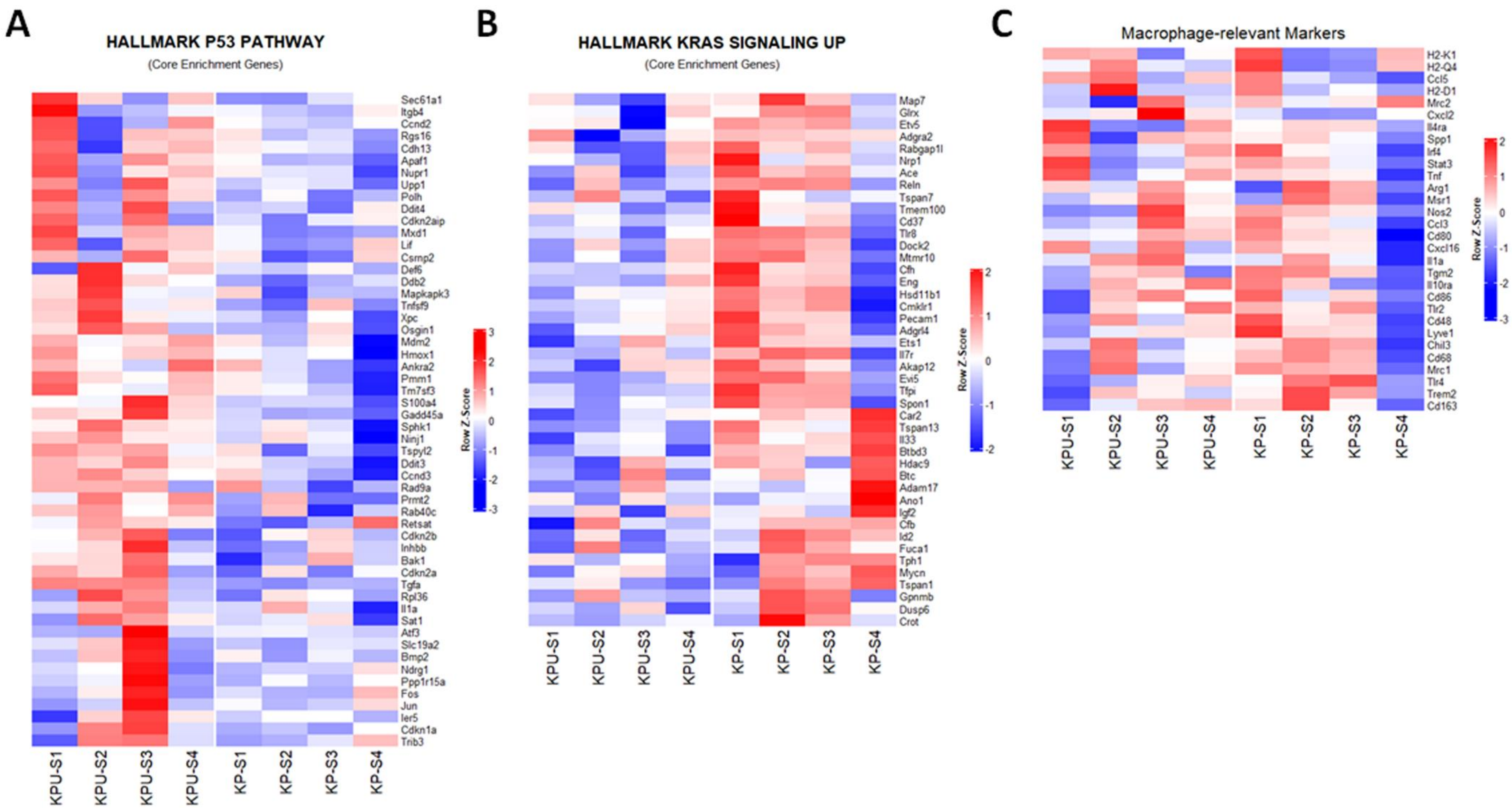

Figure S4. Unsupervised heatmaps of the expression of genes in KP and KPU- lung tumor samples (n = 4)**.** (**A**) p53 signaling pathway, (**B**) KRAS signaling pathway, and (**C**) Macrophage-relevant gene markers.

Supplementary Figure 5

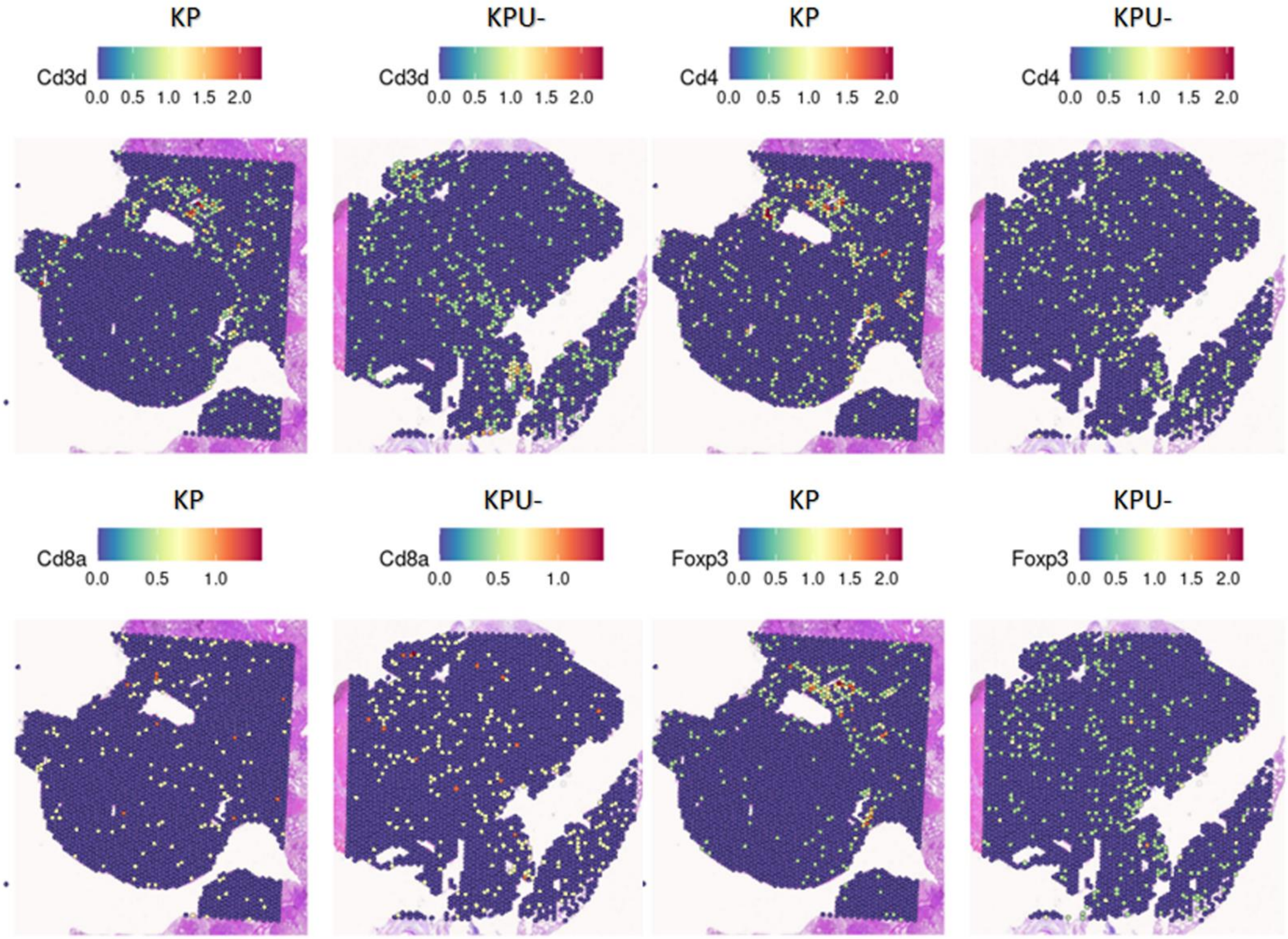

Figure S5. Spatial transcriptomic plots of Cd3d (CD3), CD4, Cd8a (CD8), and FOXP3 in a representative KP and KPU- mouse lung tumor tissues.

Supplementary Figure 6

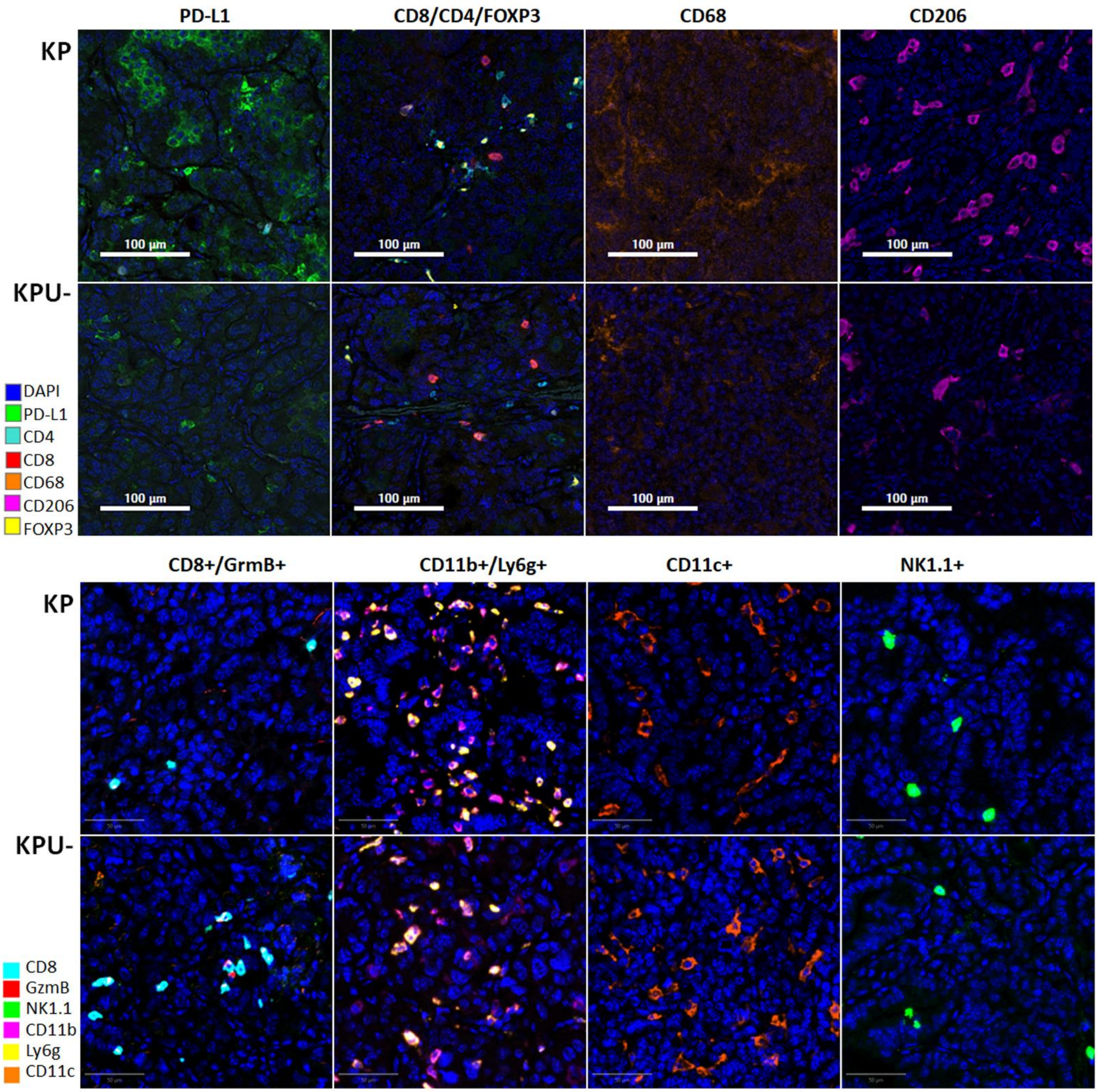

Figure S6. Representative mIF images of **PD-L1** (Green), **CD8/CD4/FOXP3** (Red/Cyan/Yellow), **CD68** (Brown), and **CD206** (purple) in upper panel); **Granzyme B** (GzmB), **NK1.1**, **CD11b**, **CD11c**, and **Ly6g** in lower panel, Scale bar: 50 µm; 200× magnification) in KP and KPU- lung tumor tissues.

Supplementary Figure 7

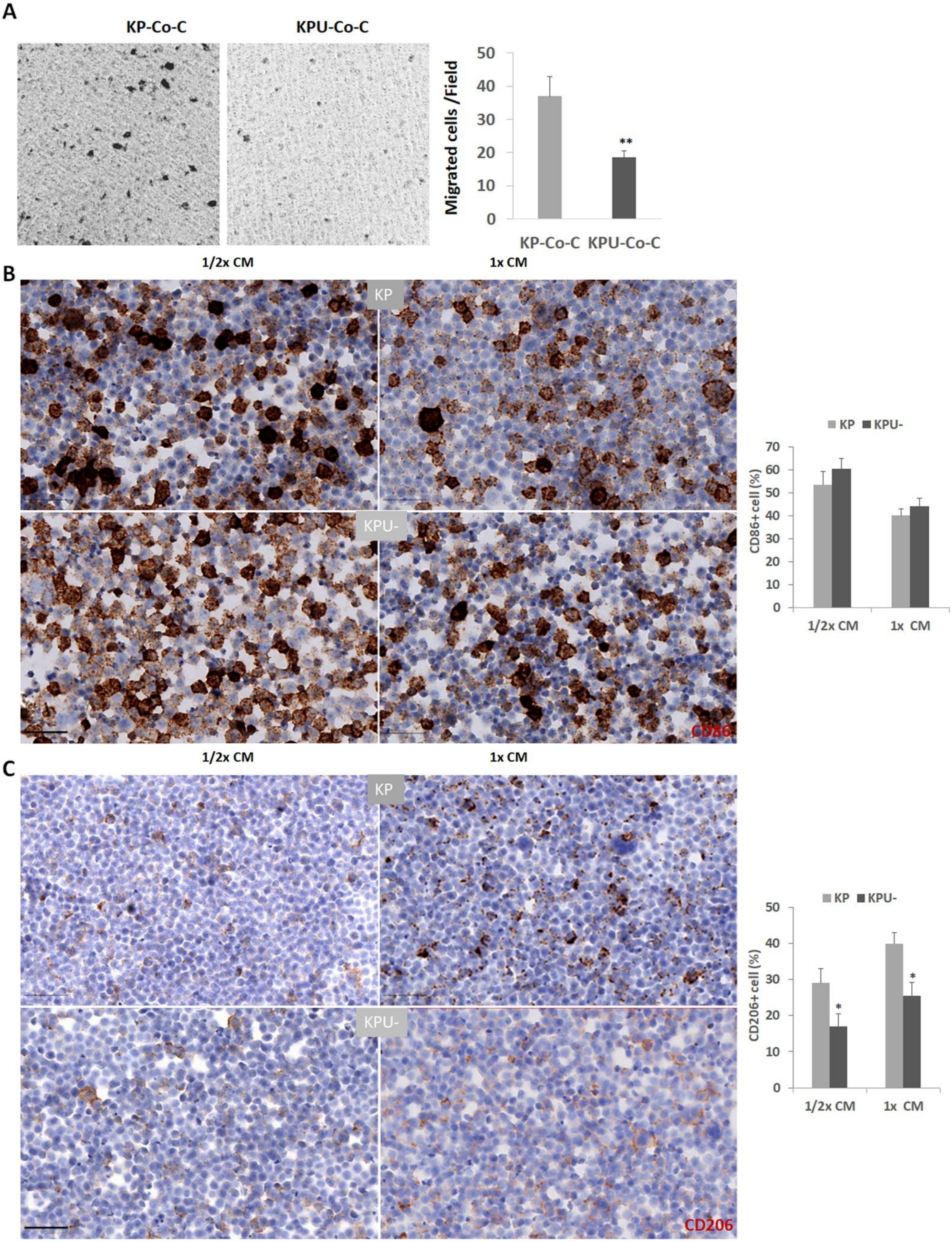

Figure S7. **Effects of KP and KPU- tumor cells on RAW264.7 macrophage migration and polarization.** (**A**) Left panel: representative images of chemotactic migration of RAW264.7 cells co-cultured with KP (KP-Co-C) or KPU- (KPU-Co-C) lung tumor cells in a Transwell assay; right panel: semiquantitative analysis of migrated cells (***P* < 0.01, KPU⁻ vs. KP). Dose-dependent suppression or enhancement of LPS/IFN-γ-induced M1 polarization (**B**) or TGF-β1-induced M2 polarization (**C**) by KP-CM and KPU-CM. Left panel: IHC staining of CD86 or CD206 (M1/M2 macrophage markers; scale bar: 50 μm; 200× magnification) in RAW264.7 cells cultured with medium containing 50% CM (1/2 CM) or 100% CM (1× CM); right panel: semiquantitative analysis of CD86- or CD206-positive cells based on data from triplicate samples from two independent experiments (**P* < 0.05, KPU vs. KP).

Supplementary Figure 8

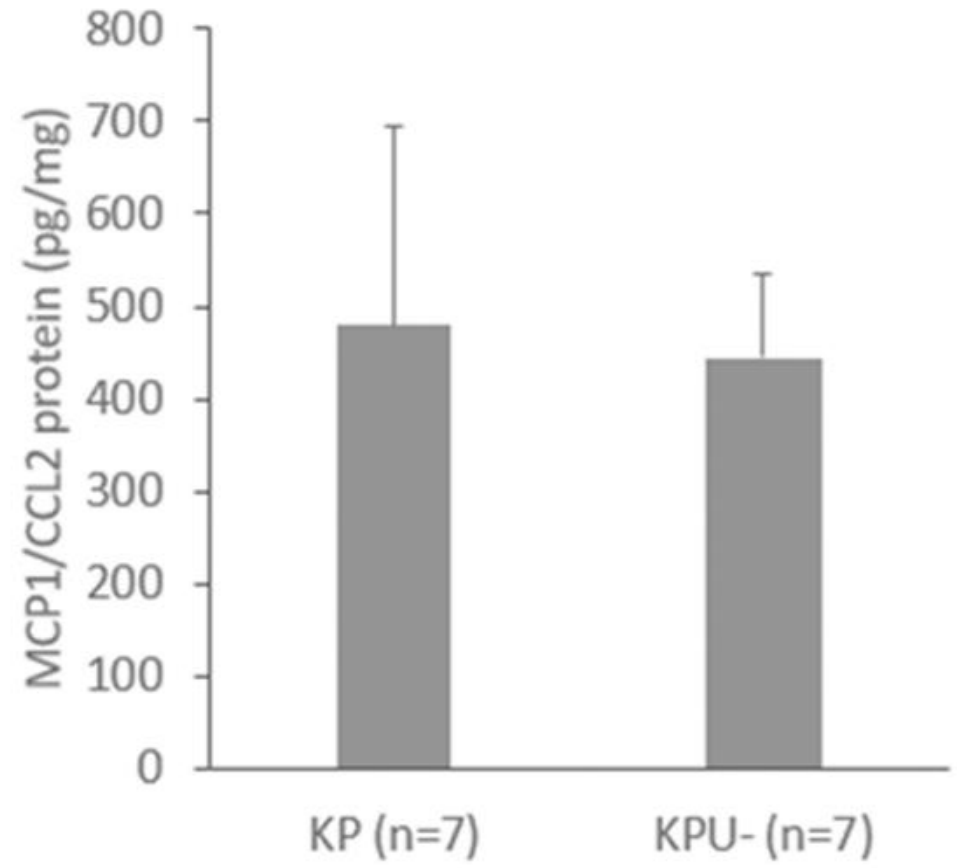

Figure S8. MCP1/CCL2 protein levels in KP and KPU- mouse lung tumors (n = 7) measured by ELISA.
